# High-throughput printing of combinatorial materials from aerosols

**DOI:** 10.1038/s41586-023-05898-9

**Published:** 2023-05-10

**Authors:** Minxiang Zeng, Yipu Du, Qiang Jiang, Nicholas Kempf, Chen Wei, Miles V. Bimrose, A. N. M. Tanvir, Hengrui Xu, Jiahao Chen, Dylan J. Kirsch, Joshua Martin, Brian C. Wyatt, Tatsunori Hayashi, Mortaza Saeidi-Javash, Hirotaka Sakaue, Babak Anasori, Lihua Jin, Michael D. McMurtrey, Yanliang Zhang

**Affiliations:** 1 Department of Aerospace and Mechanical Engineering, University of Notre Dame, Notre Dame, IN USA; 2 Department of Chemical Engineering, Texas Tech University, Lubbock, TX USA; 3 Department of Mechanical and Aerospace Engineering, University of California Los Angeles, Los Angeles, CA USA; 4 Department of Mechanical Science and Engineering, University of Illinois Urbana-Champaign, Urbana, IL USA; 5 Material Measurement Laboratory, National Institute of Standards and Technology, Gaithersburg, MD USA; 6 Department of Materials Science and Engineering, University of Maryland, College Park, MD USA; 7 Department of Mechanical and Energy Engineering and Integrated Nanosystems Development Institute, Purdue School of Engineering and Technology, Indiana University−Purdue University Indianapolis, Indianapolis, IN USA; 8 Department of Mechanical and Aerospace Engineering, California State University Long Beach, Long Beach, CA USA; 9 Idaho National Laboratory, Idaho Falls, ID USA

**Keywords:** Mechanical engineering, Materials science

## Abstract

The development of new materials and their compositional and microstructural optimization are essential in regard to next-generation technologies such as clean energy and environmental sustainability. However, materials discovery and optimization have been a frustratingly slow process. The Edisonian trial-and-error process is time consuming and resource inefficient, particularly when contrasted with vast materials design spaces^1^. Whereas traditional combinatorial deposition methods can generate material libraries^2,3^, these suffer from limited material options and inability to leverage major breakthroughs in nanomaterial synthesis. Here we report a high-throughput combinatorial printing method capable of fabricating materials with compositional gradients at microscale spatial resolution. In situ mixing and printing in the aerosol phase allows instantaneous tuning of the mixing ratio of a broad range of materials on the fly, which is an important feature unobtainable in conventional multimaterials printing using feedstocks in liquid–liquid or solid–solid phases^4–6^. We demonstrate a variety of high-throughput printing strategies and applications in combinatorial doping, functional grading and chemical reaction, enabling materials exploration of doped chalcogenides and compositionally graded materials with gradient properties. The ability to combine the top-down design freedom of additive manufacturing with bottom-up control over local material compositions promises the development of compositionally complex materials inaccessible via conventional manufacturing approaches.

## Abstract (web summary)

The authors report a high-throughput combinatorial printing method capable of fabricating materials with compositional gradients at microscale spatial resolution, demonstrating a variety of high-throughput printing strategies and applications in combinatorial doping, functional grading and chemical reaction.

## Main

Materials hold pivotal roles in many scientific and technological innovations, and progress in developing new materials is key to the pursuit of solutions to grand societal challenges. Combinatorial material depositions (for example, cosputtering) have enabled rapid screening of new materials for electronics, magnetics, optics and energy-related applications^7^. The sample-rich feature of these combinatorial material libraries facilitates elucidation of the composition–structure–property relationship and enables the rapid screening of materials over a vast range of compositions. Nevertheless, the intrinsic high-energy nature of laser or plasma excludes many materials (for example, colloidal particles, thermosensitive polymers) from use in the development of universal combinatorial material libraries. Additive manufacturing has emerged as a versatile method to fabricate materials of complex structure using micro- and nanoscale building blocks^8–10^. Recently several printing approaches, including inkjet printing, electrochemical printing and electrohydrodynamic redox printing, have been proposed for the fabrication of material libraries^11–13^. However, these methods still suffer from limited materials options and challenges in regard to the universal combination of different materials and the production of gradient material libraries, due to the lack of fast mixing mechanisms and the inability to rapidly vary mixing ratios.

**Fig. 1:**
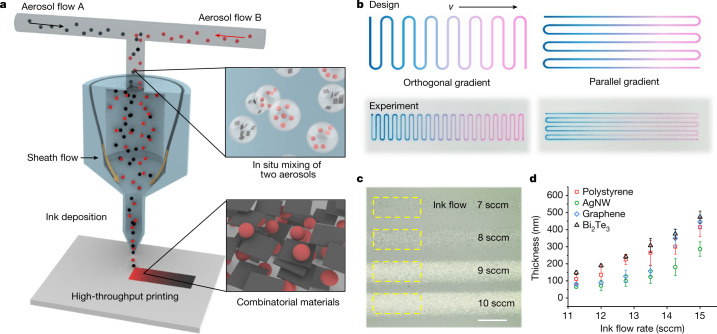
The design strategy of HTCP. **a**, Schematic illustration of the combinatorial printing method based on in situ aerosol mixing. **b**, Orthogonal and parallel gradient printing design strategies, and corresponding printed gradient patterns using blue ink (food dye Blue 1) and red ink (rhodamine B), demonstrating a compositional–modulation feature. **c**, Optical microscopy images showing the impact of aerosol ink flow rate on the deposited materials. Scale bar, 100 µm. **d**, Printed material thickness versus flow rate of various inks (polystyrene, AgNW, graphene and Bi_2_Te_3_). Error bars represent s.d. from four experimental replicates. sccm, standard cubic centimetres per minute.

For an ideal interdiffusion system, low fluid viscosity and minimal size of diffusion units are desired, which leads us to investigate the potential of using aerosols for in situ mixing and printing. Previous research on multimaterial aerosol jet printing has made steady progress in the development of functional materials and devices^14,15^, although aerosol-based printing of combinatorial gradient materials remains challenging. During aerosol-based printing, the material deposition rate can be affected by several parameters (aerosol ink flow rate, sheath gas flow rate, printing speed, atomizing voltage and so on)^16,17^, and the interplay of these printing parameters complicates aerosol mixing and deposition during printing. Unoptimized ink formulation and printing conditions may lead to unstable jetting, which can introduce uncertainty in aerosol-based printing. To understand the collective behaviour of aerosol mixing and the combinatorial printing process, we systematically investigated ink formulation, aerosol mixing and interaction, and printing parameter optimization by combining both experimental techniques (for example, fast camera imaging) and computational fluid dynamics (CFD) simulations. To achieve aerosol-based mixing and printing, our high-throughput combinatorial printing (HTCP) approach begins with atomization of two (or multiple) inks into aerosols containing microscale ink droplets, where the combined ink streams are then mixed in a single nozzle and aerodynamically focused by a co-flowing sheath gas before deposition (Fig. 1a). The aerosol jet printhead with nozzles of various sizes is applied, delivering fine features with spatial resolution as low as around 20 μm in the *x*–*y* plane and deposition thickness as low as approximately 100 nm (Supplementary Figs. 1 and 2). To generate a one-dimensional (1D) gradient material library we investigated two printing strategies—orthogonal versus parallel gradient printing (Fig. 1b). Although both approaches can generate gradient films, we found that orthogonal printing tends to be more versatile because it can tolerate a wide range of printing speeds. By contrast, a high printing speed in parallel gradient mode may lead to an undesired deposition delay that causes inaccurate ink mixing and deposition (Fig. 1b). By continuously varying the ink-mixing ratio through orthogonal printing, the compositional variation of printed materials can be achieved in a fine-gradient manner without the requirement for clean-room facilities (Supplementary Video 1 and Supplementary Table 1).

## Combinatorial mixing and printing

The printing of gradient material libraries relies on two hypotheses: (1) the controllable deposition of two materials via individual modulation of two ink flow rates; and (2) the mixing of two ink aerosols on the fly. We first evaluated the effect of ink flow rates on material deposition. As shown in Fig. 1c, material deposition rate can be controlled by adjusting ink flow rate within the stable jetting range, where the resulting deposition thicknesses of printed films increase with ink flow rate monotonically. In an optimized range of ink flow rates (Fig. 1d), we found that this monotonic trend can be applied to a variety of nanomaterial inks including Ag nanowires (AgNW), graphene, Bi_2_Te_3_ and polystyrene, although an extremely high aerosol flow rate may lead to unstable jetting^17,18^. Other printing parameters have been systematically investigated to optimize printing processes and realize high printing reproducibility and stability. For printing and ink parameters see Supplementary Fig. 3 and Supplementary Tables 2–5; for ink priming, Supplementary Fig. 4; for printing stability, Supplementary Fig. 5; for batch-to-batch reproducibility, Supplementary Fig. 6; for printing uncertainty, Supplementary Fig. 7; for correlation matrix of parameters, Supplementary Fig. 8; for aerosol ink flow range, Supplementary Fig. 9; for ink effect on surface smoothness, Supplementary Fig. 10; for printing reproducibility of gradient samples, Supplementary Figs. 11 and 12; for substrate effect, Supplementary Figs. 13 and 14; and for typical flow, Supplementary Fig. 2a. In particular, we analysed the role of printing process variables to show the relationship between process variability and underlying mechanisms. To minimize process variability due to changes in ink properties, ink formulation (for example, solvents and surfactants) needs to be engineered to ensure long-term chemical stability^19^ and colloidal stability^20^. It is also important to optimize printing process parameters to control the key flow characteristics and ensure stable jetting with low variability for aerosol-based printing. In addition, fast camera imaging was utilized to understand the behaviour of aerosol jetting of inks, where we observed the effect of Saffman force on aerosol droplets with a strong collimating effect that helps narrow the passage of ink aerosols (for sheath flow effect see Supplementary Fig. 15 and Supplementary Video 2; for time-dependent jetting, Supplementary Fig. 16; and for aerosols of fluorescent inks, Supplementary Fig. 17). Furthermore, CFD simulations were conducted to understand the underlying aerosol-based ink-mixing mechanisms under different sheath gas flow conditions. CFD analyses, combined with mixing index calculations, show considerable enhancement in the mixing of two aerosol ink flows when increasing sheath gas flow rates (for CFD model see Supplementary Fig. 20; for ink mixing versus sheath flow, Supplementary Fig. 21; for mixing index and flow profile, Supplementary Figs. 22 and 23; and for simulation parameters, Supplementary Table 6), which is consistent with our experimental observations from fast camera imaging. This is an important finding because it provides a previously unexplored avenue to dynamically control ink mixing on the fly using sheath gas flow-induced aerodynamic focusing without the need for a complicated external mixer. In addition, CFD results indicate that a smaller nozzle diameter can enhance the convergence and mixing of aerosols (Supplementary Fig. 24), although an overly small nozzle (under 50 µm) may increase the probability of clogging during the printing process.

**Fig. 2:**
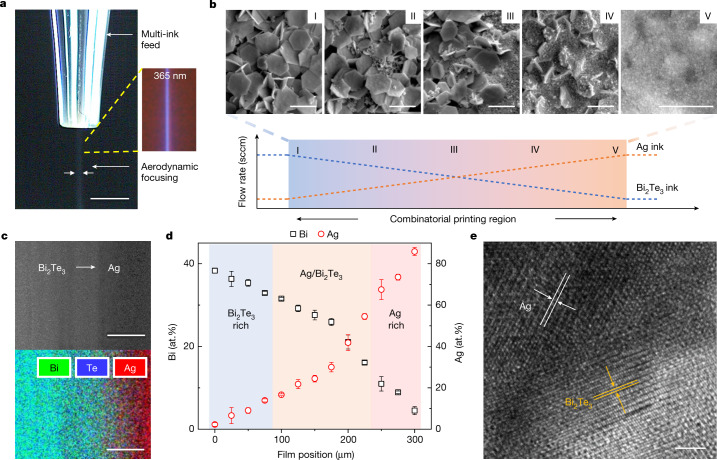
Rapid printing of combinatorial materials with gradient compositions. **a**, Fast camera image showing the aerosol deposition process of photoluminescent inks (blue) under visible light and ultraviolet light of 365 nm wavelength. Scale bar, 2.5 mm. **b**, Typical gradient profile of the HTCP process. SEM images demonstrate the morphology changes in Ag/Bi_2_Te_3_ composites with increasing Ag ink flow and decreasing Bi_2_Te_3_ ink flow. The fabrication process takes approximately 1 min. Scale bars, 1 µm. **c**, Compositional characterization of Ag/Bi_2_Te_3_ composites with SEM (top) and EDS (bottom). Scale bars, 100 µm. **d**, Elemental distributions of Ag/Bi_2_Te_3_ composites over gradient direction. Error bars represent s.d. from three gradient samples. **e**, TEM of Ag/Bi_2_Te_3_ composites showing the interface between Ag nanoparticles and Bi_2_Te_3_ nanoplates. Scale bar, 2 nm.

As a demonstration of the HTCP process, we printed a metal/semiconductor nanocomposite using two inks containing zero-dimensional (0D) Ag nanoparticles of around 60 nm in diameter and two-dimensional (2D) Bi_2_Te_3_ nanoplates of around 1 µm in lateral size. Once a narrow stream of mixed aerosols was formed by the optimized aerodynamic focusing, we gradually increased the metal-to-semiconductor ink-mixing ratio and observed a clear morphological transition from a Bi_2_Te_3_ nanoplate-rich phase to a well-blended composite phase and then to a Ag nanosphere-rich phase (Fig. 2b). Changes in chemical composition were confirmed by energy-dispersive X-ray spectroscopy (EDS), as shown in Fig. 2c. A clearly increasing trend of Ag content in the printed combinatorial film was observed (Fig. 2d). Similar to the cosputtering method^1^, HTCP is not aimed at generation of strictly linear-gradient compositions in combinatorial materials; instead, it is intended to produce gradient sample features in a fast, monotonic and high-throughput manner. Whereas certain process variations exist in HTCP printing, as indicated by the error bars in EDS measurements, a monotonic compositional gradient and distinct compositions of roughly 25 μm spatial resolution are observed along the gradient printing direction (elemental distribution, Fig. 2d; sample-to-sample reproducibility, Supplementary Fig. 18; transmission electron microscopy (TEM) of Ag/Bi_2_Te_3_ showing chemically fused nanocomposites after thermal sintering, Fig. 2e; TEM–EDS analysis, Supplementary Fig. 19).

In general, composite fabrications involve a process that mixes one or several filler materials into a matrix material to achieve synergistic properties. A conventional trial-and-error approach often requires extensive processing time, which not only causes difficulty with high-throughput fabrication but also may lead to undesired side reactions from the mismatch of starting materials related to their surface charge, pH values and ionic strength^21,22^. For example, MXene and Sb_2_Te_3_ nanoparticles exhibit opposite surface charges in a certain pH range^23^, leading to the formation of large aggregates with poor colloidal stability (Supplementary Fig. 25). By contrast, the HTCP technique enables the rapid fabrication of combinatorial samples with gradient compositions, which minimizes undesired side effects (for example, aggregation). Aerosol-based HTCP uses nitrogen as the carrying gas, which forms a protective ‘gas barrier’ between ink droplets such that MXene and Sb_2_Te_3_ will not interact/react until being deposited onto the desired location of substrates. Consequently a dense, uniform composite film of MXene/Sb_2_Te_3_ was successfully printed (Supplementary Fig. 26). Thus, the combinatorial printing of seemingly incompatible materials is particularly unique and different from previous printing methods.

**Fig. 3:**
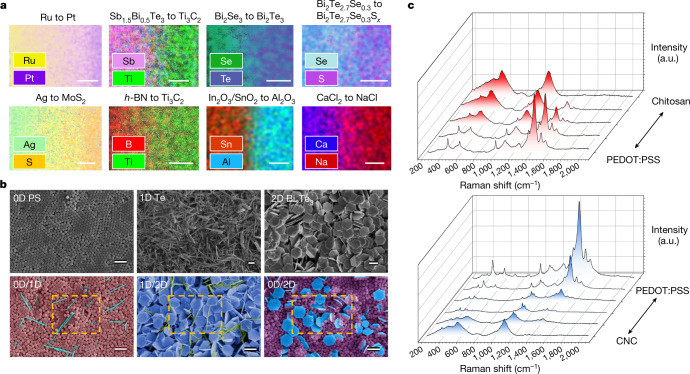
HTCP with broad range of material options. **a**, Elemental mapping of various printed combinatorial systems covering a broad range of elements. Scale bars, 300 µm. **b**, SEM images of various combinatorial materials printed directly using 0D, 1D and 2D nanoparticles. Scale bars, 1 μm. **c**, Combinatorial printing of polymers. Raman spectra confirm the compositional shift in combinatorial PEDOT:PSS/chitosan (top) and CNC/PEDOT:PSS (bottom). a.u., arbitrary units.

To explore the full capacity of the HTCP method, we fabricated a wide spectrum of films with gradient compositions including metals, oxides, nitrides, carbides, chalcogenides and halides, containing elements from s-block (groups IA–IIA) and p-block (groups IIIA–VIIA) of the periodic table (Fig. 3a). We also printed inks containing several d-block elements into combinatorial material libraries, including transition metal chalcogenides (for example, MoS_2_) and transition metal carbides (for example, MXenes) (Supplementary Fig. 27). Moreover, the HTCP approach also exhibits excellent tolerance to material dimensions and morphology, as demonstrated in a printed 0D/1D composite of polystyrene (PS)/Te nanowires, a 1D/2D composite of Te nanowires/Bi_2_Te_3_ nanoplates and a 0D/2D composite of PS/Bi_2_Te_3_ nanoplates (scanning electron microscopy (SEM) images, Fig. 3b). SEM analyses on fractured cross sections of 0D/2D composite of Ag/Bi_2_Te_3_ reveal a homogeneous distribution of nanoscale building blocks without phase separations (Supplementary Fig. 28). In addition to inorganic nanomaterials, temperature-sensitive polymers (including biopolymers and semiconducting polymers) were tested (Fig. 3c). The Raman spectra of combinatorial polymer films of chitosan and poly(3,4-ethylenedioxythiophene)-poly(styrenesulfonate) (PEDOT:PSS) show a peak evolution at approximately 1,094 cm^−1^ for chitosan^24^ and at approximately 1,434 cm^−1^ for PEDOT:PSS^25^, indicating a clear compositional transition from chitosan to PEDOT:PSS. Similarly, cellulose nanocrystals (CNC) and PEDOT:PSS were printed into a gradient material library and the compositionally varying feature was verified using Raman analysis. These results demonstrate the versatility of HTCP in the rapid fabrication of a broad range of inorganic and organic combinatorial materials, substantially expanding the material options for high-throughput additive manufacturing.

## HTCP of functional systems

**Fig. 4:**
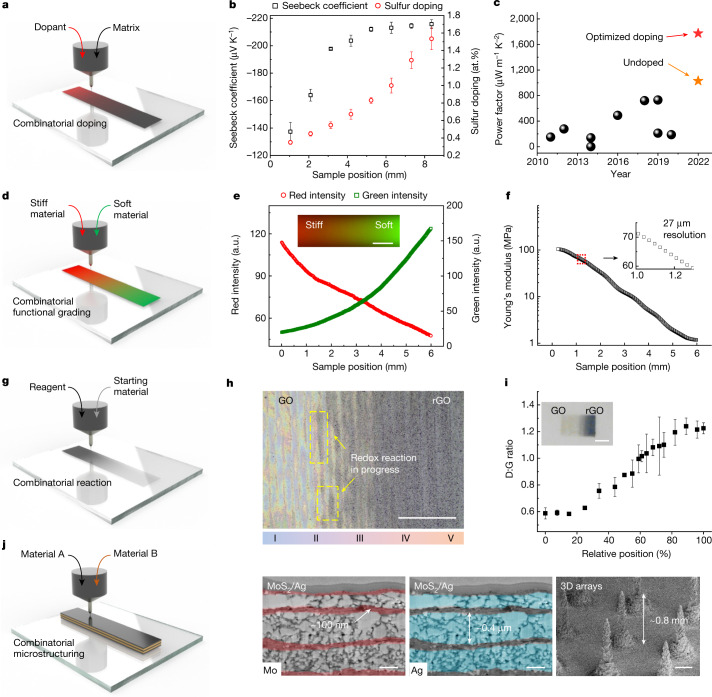
HTCP enables combinatorial doping, functional grading, chemical reaction and compositional microstructuring. **a**, Schematic of combinatorial doping. **b**, A Bi_2_Te_2.7_Se_0.3_ film with gradient sulfur doping concentrations and resulting local Seebeck coefficient changes. Error bars represent s.d. from two experimental replicates for Seebeck coefficient and six experimental replicates for sulfur doping concentration. **c**, Room temperature power factor comparisons between our printed Bi_2_Te_2.7_Se_0.3_ with optimized doping (red) and other printed n-type materials over the past decade^26–32^, where our undoped Bi_2_Te_2.7_Se_0.3_ is coloured orange for reference. **d**, Schematic of combinatorial printing of functionally graded materials. **e**, Fluorescent image showing red and green light intensities of a gradient polyurethane film printed with two PUD inks coloured by two dyes, with the inset showing a photoluminescent image. Scale bar, 1 mm. **f**, Local Young’s modulus versus sample position measured with spatial resolution of about 27 µm. **g**, Schematic of combinatorial chemical reaction. **h**, Optical microscopic image of a GO/rGO gradient film. Scale bar, 0.5 mm. **i**, Raman analysis showing the D:G band ratio at different sample locations, with the inset showing an optical image of a gradient film following overnight reaction. Scale bar, 1 mm. Error bars represent s.d. from three experimental replicates. **j**, Left, schematic of combinatorial microstructuring. Right, examples of Ag/MoS_2_ with periodic structures of submicron resolution along the thickness direction (false-coloured SEM showing Mo in red and Ag in blue; scale bars, 300 nm) and printed 3D arrays of salt crystals (scale bar, 300 μm).

To explore the potential of using the printed material library to accelerate materials screening and optimization toward the desired properties, we demonstrated a high-throughput combinatorial doping strategy for thermoelectric applications (Fig. 4a). Due to high scalability and design freedom, thermoelectric (TE) printing has been extensively pursued in the past decade in the development of flexible or 3D conformal devices for energy harvesting and cooling. However, the relatively low performance (typically 10^2^–10^3^ µW m^–1^ K^–2^ in power factor^26–32^) of printed n-type materials has thwarted the prospect of realizing broad impacts of printed thermoelectrics. To improve printed n-type materials, HTCP was used for rapid optimization of sulfur doping concentrations in printed Bi_2_Te_2.7_Se_0.3_ materials, where samples of gradient doping concentrations were printed and tested in a single combinatorial gradient film. With increasing sulfur doping concentration, the Seebeck coefficient of printed Bi_2_Te_2.7_Se_0.3_ film sharply increased from −130 to −200 µV K^–1^ (at about 0.5% S) and then reached a plateau at around −213 µV K^–1^ (at about 1.0% S; Fig. 4b). Such profound change could have originated from the increased density of states (DOS) effective mass ${m}_{{\rm{DOS}}}^{* }$ enabled by sulfur doping^33^. The combinatorial doping of a Bi_2_Te_2.7_Se_0.3_ film shows a peak thin-film power factor at an optimal sulfur doping concentration of about 1.0 atomic weight percentage (at.%) (TE property versus doping; Supplementary Fig. 29). This printed material library is primarily aimed at identification of the optimized doping composition as opposed to achieving absolute property values. Because the electrical conductivity of the aerosol jet-printed films is limited by the requirement of using low-viscosity inks with relatively low particle concentration, an extrusion printing technique was applied to convert highly concentrated Bi_2_Te_2.7_Se_0.3_ inks to thick films for practical device applications. Subsequent TE property measurements show a maximum room temperature power factor of 1,774 µW m^–1^ K^–2^ at 1.0% sulfur doping (Supplementary Fig. 30), which is substantially higher than most printed n-type TE materials (Fig. 4c and Supplementary Table 7)^26–32^. From a fundamental perspective, we also explored the combinatorial feature of HTCP to understand the compositional effect on Seebeck coefficient and charge carrier transport behaviour (ternary Sb_x_Bi_2-x_Te_3_ and quaternary Sb_x_Bi_(0.3x+6.7y)_Te_(2x+9y)_Se_y_ alloys; Supplementary Fig. 31). These results demonstrate the data-rich feature of HTCP in efficient identification of optimized material compositions for achieving the desired properties.

In addition to material screening, we explored the potential of HTCP in the fabrication of functionally graded materials. As proof of concept, we printed gradient polyurethane films using two polyurethane dispersion (PUD) inks with different elastic moduli (Fig. 4d). The resultant gradient mixing can be visualized via fluorescent imaging by incorporation of the two PUD inks with red and green fluorescent dye, respectively (Fig. 4e, inset). A subsequent red, green and blue analysis of the functionally graded polyurethane (FGP) shows a monotonic trend of the compositional gradient with fluorescent intensity measured every 22.8 μm over the entire 6-mm-long film (Fig. 4e). To measure the mechanical property of the FGP, a tensile test accompanied by the 2D digital image correlation (DIC) method was used to map the strain field, and thereby to obtain the distribution of Young’s modulus with a spatial resolution of around 27 μm. As shown in Fig. 4f, with increasing mixing ratio of soft to stiff PUD inks, FGP shows a monotonic decrease in Young’s modulus over two orders of magnitude (from 103 to 1.2 MPa). Materials with such a gradient modulus may cover a range of biomaterials (tendon, skin, muscle and so on)^34^ and may find applications in such as interface materials between components of different mechanical properties (Young’s modulus versus biomaterials, Supplementary Fig. 32; stretchability test, Supplementary Fig. 33). These results indicate the ability of HTCP to achieve monotonic gradation of both compositions and properties at 20–30 μm spatial resolution.

Besides printing stable inks, we investigated HTCP using reactive inks and their combinatorial reaction behaviours (Fig. 4g). During this process, reactive ink materials may undergo chemical/biochemical reactions triggered by the convergence of two inks and/or stimuli such as light, heat or catalysts. As proof of concept, graphene oxides (GOs) were coprinted with ascorbic acid (AC) under a gradient mixing ratio in which AC reduces GO to reduced graphene oxide (rGO). As the reaction proceeds, it is evident that a higher AC concentration causes a more rapid change in GO colour, from light yellow to dark brown, in the AC-rich region (Fig. 4h). Once the gradient-reduction reaction had reached completion, the inset of Fig. 4i shows the combinatorial film with a gradient appearance from light yellow (GO) to black (rGO). Raman spectroscopy (Fig. 4i and Supplementary Fig. 34) shows a clear change in D:G band ratio with increasing AC ink flow. This indicates that the reduction in GO decreased the average size of the sp^2^ domains, because new graphitic domains were created with smaller sizes and larger quantities compared with those in the unreduced GO^35^. The HTCP method can also enable heterogenous fabrication of materials with compositionally complex structures by alternately depositing two ink materials layer by layer, leading to combinatorial microstructuring (Fig. 4j). An advantage of aerosol-based ink deposition is the ability to quickly switch from one material to the other due to the low viscous drag (for example, the Ag/MoS_2_ nanocomposite with periodic structures shown in Fig. 4j), achieving submicron spatial resolution (roughly 100 nm) along film thickness direction, which is difficult to realize using other multimaterial printing methods (for example, extrusion printing). The Ag and MoS_2_ inks for compositional microstructuring were formulated to be immiscible, to minimize diffusion between different layers. The microstructure patterning can also be applied to achieve 3D structures with a high aspect ratio (for example, the 3D pillars shown in Fig. 4j).

## Conclusions

The HTCP method enables high-throughput fabrication of versatile material libraries with gradient compositions utilizing rapid aerosol-based mixing and modulation of the mixing ratio. This in situ mixing and printing method may spark multiple potential research directions. First, HTCP can fabricate gradient films of metals, nitrides, carbides, chalcogenides, halides and even seemingly incompatible materials, enabling combinatorial materials screening and optimization with vastly expanded material options. Second, HTCP can produce functionally graded materials with unique compositional/structural arrangements and superior properties surpassing their constitutive materials with homogenous compositions. In addition, the combinatorial printing of reactive materials offers new possibilities toward high-throughput exploration, experimentation and characterization of chemicals/materials syntheses. The next phase of research will focus on leveraging the fabrication freedom and data-rich nature of HTCP, along with machine learning- and artificial intelligence-guided design strategies, which are expected to accelerate the discovery and development of a broad range of materials with intriguing and unprecedented properties for emerging applications.

## Methods

### Nanoparticle synthesis

Colloidal nanoparticles were prepared via either bottom-up synthesis or top-down exfoliation. For example, metal chalcogenides were synthesized via the bottom-up methods reported in our previous work^30,36^. Taking Sb_2_Te_3_ synthesis for example, a mixture of ethylene glycol and diethylene glycol in a proportion of 1:2, with a volume of 150 ml, was combined with 12 mmol of SbCl_3_, 18 mmol of TeO_2_, 3 g of NaOH and 0.8 g of polyvinylpyrrolidone with a molecular weight of 40,000 g mol^–1^. The mixture was heated under reflux at 190 °C for 15 h and the precipitates separated by centrifugation at 5,000 rpm. To remove any remaining impurities, the precipitates were washed with ethanol three times. Similarly, Bi_2_Te_3_ synthesis was achieved using different metal precursors (for example, bismuth(III) nitrate pentahydrate). The Ti_3_C_2_T_*x*_ MXene nanosheets (T represents surface termination, such as −O, −OH or −F) were synthesized by selective etching of aluminium from the MXene precursor, Ti_3_AlC_2_, using a combined hydrofluoric acid/hydrochloric acid mixture and lithium chloride for delamination, as described previously^37^. For carbon-based nanosheets, liquid-phase exfoliation methods were used. Additional details on material synthesis are available in the Supplementary Information.

### General ink formulation

In a typical aqueous ink formulation a mixed solvent of water and ethylene glycol was used to disperse nanoparticles, in which ethylene glycol serves as a cosolvent to improve ink stability and printability. Depending on the ink type, a small amount of isopropyl alcohol may also be added as a defoamer to suppress the formation of foam during the ultrasonic atomizing process. To avoid aggregation and ensure uniform dispersion of the inks, they were sonicated for 15 min (Hilsonic bath sonicator, 300 W). Typical ink composition can be found in Supplementary Table 2. The concentration of nanomaterial was determined by the drying weight method. Commercially available inks, including indium tin oxides (30 wt.% in isopropanol), molybdenum disulfide (in terpineol/cyclohexanone) and aluminium oxide nanoparticle (20 wt.% in H_2_O), were purchased from Sigma-Aldrich. Silver nanoparticle ink (PRELECT TPS 50G2) was obtained from Clariant Specialty Chemicals. For nonparticle inks, molecular or salt precursors were directly dissolved in solvents before the printing processes.

### HTCP printing

The motion control graphical user interface (GUI) controls the *x*, *y*, *z* motion stages of the printer. Real-time position and velocity were tracked for *x*, *y*, *z* during the targeted move and jogging operations. Both aerosol ink flow rates were actively controlled with respect to stage position to achieve gradient and voxelated films. For all high-resolution printing, the inks were first primed under sonication for 30 min and then atomized via ultrasonication before transfer to the printhead. A sheath flow was used to focus the aerosolized ink stream to achieve high printing resolution. Before printing, substrates (such as glass, mica, Kapton and so on) were precleaned with isopropyl alcohol and treated with plasma to improve surface hydrophilicity. During the HTCP process, a heating stage was used to evaporate the ink solvents to minimize undesired drying effects. Depending on the type of combinatorial materials, additional thermal sintering was adopted to achieve the desired microstructures and properties.

### Thermoelectric property characterization

The Seebeck coefficient and electrical conductivity were measured using a custom-built scanning probing system consisting of two fine 40 AWG k-type thermocouples placed about 1 mm apart and a heater placed about 1 mm away from one of the thermocouples. Two electrodes through which current was sent for electrical conductivity measurement were placed at both ends of the printed film. At each measurement location, electrical conductivity was measured based on a linear four-probe principle at thermal equilibrium before measuring the Seebeck coefficient. During Seebeck coefficient measurement, heating power was slowly increased and Seebeck voltage between the two thermocouples was collected continuously along with thermocouple temperatures. The absolute Seebeck coefficient of the film was calculated by taking into account the Seebeck coefficient of the thermocouple wire. The measurement system was validated by measuring a bulk constantan film with known thermoelectric properties. In addition, the printed gradient films were also measured using a custom-built scanning probe instrument at the National Institute of Standards and Technology^38^. The Seebeck coefficient was measured in 0.5 mm increments using a pair of independently spring-loaded type R thermocouple probes spaced 3 mm apart, using the quasisteady state condition of the differential method^39^. Measurement uncertainty (1 s.d.) for Seebeck measurement is ±6.5%. Details of these thermoelectric measurements can be found in Supplementary Information.

### Mechanical property mapping of gradient polyurethane films

To obtain Young’s modulus distribution of a printed gradient polyurethane film, we used the 2D DIC method to track strain and displacement distributions under uniaxial stretch. The specimen had a length of 12 mm, width of 2 mm and a gradient composition. The force–displacement relation was recorded by uniaxial stretching of the specimen up to 50% strain at a rate of 0.1% s^–1^ via an Instron universal machine (model 5944) with a 50 N load cell. The specimen was mounted in a pair of tensile grips, leaving a gauge length of 8 mm. The ratio of length to width (4) is sufficiently high to ensure that the majority of the specimen undergoes uniaxial tension with negligible edge effects. To measure strain distribution by the DIC method we sprayed ink (Koh-I-Noor Rapidraw) with an airbrush (Badger, no. 150) to generate high-quality speckle patterns on the specimen. To enhance optical contrast, a whiteboard was used as background and a white light LED light was shot on the sample during testing. Changes in speckle patterns were recorded by a Canon ESO 6D DSLR camera with a Canon 100 mm F/2.8L macro lens at roughly every 1% strain. The resolution of each image was around 6.8 μm per pixel, and we output data every four-pixel length. Images were analysed by Ncorr^40^, an open-source 2D DIC Matlab software, to obtain the strain and displacement distributions of the middle region of 6 mm (L) × 2 mm (W). We tested the specimen four times to eliminate natural error and, after each loading and unloading, the specimen was placed on a hotplate at 35 °C for 5 min, and at room temperature for a further 10 min, to fully release any residual stress. Based on the measured strain distribution, we calculated Young’s modulus distribution in the elongation direction under the assumption of linearly elastic material and no gradient modulus along the width (Supplementary Information).

## Online content

Any methods, additional references, Nature Portfolio reporting summaries, source data, extended data, supplementary information, acknowledgements, peer review information; details of author contributions and competing interests; and statements of data and code availability are available at 10.1038/s41586-023-05898-9.

## Supplementary information

Supplementary Information Supplementary Information containing Methods, Figs. 1–34 and Tables 1–7; see Contents page for details.

Supplementary Video 1 Demonstration of real-time HTCP process for the printing of gradient combinatorial materials, in which a gradient-coloured film was fabricated by shifting the mixing ratio of blue and yellow dye inks on the fly.

Supplementary Video 2 Typical aerosol jet-printing process for HTCP at different sheath flow rates. Despite the challenge of optical imaging on aerosol streams, it was observed that a higher sheath flow leads to a narrower aerosol stream. The video was taken using a high-speed camera under a frame rate of 5,000 s^–1^.

## Data Availability

The datasets generated or analysed during the current study are available from the corresponding author on request.

## References

[1] Ludwig A (2019). Discovery of new materials using combinatorial synthesis and high-throughput characterization of thin-film materials libraries combined with computational methods. NPJ Comput. Mater..

[2] McGinn PJ (2019). Thin-film processing routes for combinatorial materials investigations—a review. ACS Comb. Sci..

[3] Xiang X-D (1995). A combinatorial approach to materials discovery. Science.

[4] Lopes LR, Silva AF, Carneiro OS (2018). Multi-material 3D printing: the relevance of materials affinity on the boundary interface performance. Addit. Manuf..

[5] Skylar-Scott MA, Mueller J, Visser CW, Lewis JA (2019). Voxelated soft matter via multimaterial multinozzle 3D printing. Nature.

[6] Xing X (2020). High-resolution combinatorial patterning of functional nanoparticles. Nat. Commun..

[7] Green ML, Takeuchi I, Hattrick-Simpers JR (2013). Applications of high throughput (combinatorial) methodologies to electronic, magnetic, optical, and energy-related materials. J. Appl. Phys..

[8] Jiang C (2019). Printed subthreshold organic transistors operating at high gain and ultralow power. Science.

[9] Kim Y, Yuk H, Zhao R, Chester SA, Zhao X (2018). Printing ferromagnetic domains for untethered fast-transforming soft materials. Nature.

[10] Saccone MA, Gallivan RA, Narita K, Yee DW, Greer JR (2022). Additive manufacturing of micro-architected metals via hydrogel infusion. Nature.

[11] Chen X (2019). Multi-metal 4D printing with a desktop electrochemical 3D printer. Sci. Rep..

[12] Reiser A (2019). Multi-metal electrohydrodynamic redox 3D printing at the submicron scale. Nat. Commun..

[13] Queraltó A (2021). Combinatorial screening of cuprate superconductors by drop-on-demand inkjet printing. ACS Appl. Mater. Interfaces.

[14] Wang K, Chang Y-H, Zhang C, Wang B (2016). Conductive-on-demand: tailorable polyimide/carbon nanotube nanocomposite thin film by dual-material aerosol jet printing. Carbon.

[15] Craton MT, Albrecht JD, Chahal P, Papapolymerou J (2021). Multimaterial aerosol jet printed magnetic nanocomposites for microwave circuits. IEEE Trans. Compon. Packaging Manuf. Technol.

[16] Mahajan A, Frisbie CD, Francis LF (2013). Optimization of aerosol jet printing for high-resolution, high-aspect ratio silver lines. ACS Appl. Mater. Interfaces.

[17] Tafoya RR (2020). Real-time optical process monitoring for structure and property control of aerosol jet printed functional materials. Adv. Mater. Technol..

[18] Zhang H, Moon SK, Ngo TH (2019). Hybrid machine learning method to determine the optimal operating process window in aerosol jet 3D printing. ACS Appl. Mater. Interfaces.

[19] Zeng M (2021). Scalable nanomanufacturing of chalcogenide inks: a case study on thermoelectric V–VI nanoplates. J. Mater. Chem. A.

[20] Zeng M (2020). Colloidal nanosurfactants for 3D conformal printing of 2D van der Waals materials. Adv. Mater..

[21] Bouyer F, Robben A, Yu WL, Borkovec M (2001). Aggregation of colloidal particles in the presence of oppositely charged polyelectrolytes: effect of surface charge heterogeneities. Langmuir.

[22] Lin W (2006). Heteroaggregation in binary mixtures of oppositely charged colloidal particles. Langmuir.

[23] Lu X (2020). High-efficiency thermoelectric power generation enabled by homogeneous incorporation of MXene in (Bi,Sb)2Te3 matrix. Adv. Energy Mater..

[24] Souza NLGD, Brandão HM, de Oliveira LFC (2011). Spectroscopic and thermogravimetric study of chitosan after incubation in bovine rumen. J. Mol. Struct..

[25] Lee HJ (2016). Enhanced thermoelectric performance of PEDOT:PSS/PANI–CSA polymer multilayer structures. Energy Environ. Sci..

[26] We JH, Kim SJ, Cho BJ (2014). Hybrid composite of screen-printed inorganic thermoelectric film and organic conducting polymer for flexible thermoelectric power generator. Energy.

[27] Chen A, Madan D, Wright PK, Evans JW (2011). Dispenser-printed planar thick-film thermoelectric energy generators. J. Micromech. Microeng..

[28] Madan D (2012). Enhanced performance of dispenser printed MA n-type Bi2Te3 composite thermoelectric generators. ACS Appl. Mater. Interfaces.

[29] Kim F (2018). 3D printing of shape-conformable thermoelectric materials using all-inorganic Bi2Te3-based inks. Nat. Energy.

[30] Saeidi-Javash M, Kuang W, Dun C, Zhang Y (2019). 3D conformal printing and photonic sintering of high-performance flexible thermoelectric films using 2D nanoplates. Adv. Funct. Mater..

[31] Ferhat S (2019). Flexible thermoelectric device based on TiS2(HA)x n-type nanocomposite printed on paper. Org. Electron..

[32] Su N, Zhu P, Pan Y, Li F, Li B (2020). 3D-printing of shape-controllable thermoelectric devices with enhanced output performance. Energy.

[33] Devender (2016). Harnessing topological band effects in bismuth telluride selenide for large enhancements in thermoelectric properties through isovalent doping. Adv. Mater..

[34] McKee CT, Last JA, Russell P, Murphy CJ (2011). Indentation versus tensile measurements of Young’s modulus for soft biological tissues. Tissue Eng. Part B Rev..

[35] Stankovich S (2007). Synthesis of graphene-based nanosheets via chemical reduction of exfoliated graphite oxide. Carbon.

[36] Dun C (2019). 3D printing of solution-processable 2D nanoplates and 1D nanorods for flexible thermoelectrics with ultrahigh power factor at low-medium temperatures. Adv. Sci..

[37] Saeidi-Javash M (2021). All-printed MXene–graphene nanosheet-based bimodal sensors for simultaneous strain and temperature sensing. ACS Appl. Electron. Mater..

[38] Yan YG, Martin J, Wong-Ng W, Green M, Tang XF (2013). A temperature dependent screening tool for high throughput thermoelectric characterization of combinatorial films. Rev. Sci. Instrum..

[39] Martin J (2013). Protocols for the high temperature measurement of the Seebeck coefficient in thermoelectric materials. Meas. Sci. Technol..

[40] Blaber J, Adair B, Antoniou A (2015). Ncorr: open-source 2D digital image correlation Matlab software. Exp. Mech..

