# Supplementary material for: High-throughput printing of combinatorial materials from aerosols
Source: Nature. 2023 May 10;617(7960):292–8. doi: 10.1038/s41586-023-05898-9 (PMC10172128; doi:10.1038/s41586-023-05898-9)
Supplement: Supplementary file 1 — Supplementary Information containing Methods, Figs. 1–34 and Tables 1–7; see Contents page for details. [file 41586_2023_5898_MOESM1_ESM.pdf]

nature portfolio

https://doi.org/10.1038/s41586-023-05898-9

Supplementary information

High-throughput printing of combinatorial materials from aerosols

In the format provided by the authors and unedited

Supplementary Information

High-throughput printing of combinatorial materials from aerosols

Minxiang Zeng,[1,2,10] Yipu Du,[1,10] Qiang Jiang,[1,10] Nicholas Kempf,[1] Chen Wei,[3] Miles V. Bimrose,[1,4] A.N.M. Tanvir,[1] Hengrui Xu,[1] Jiahao Chen,[1] Dylan J. Kirsch,[5,6] Joshua Martin,[5] Brian C. Wyatt,[7] Tatsunori Hayashi,[1] Mortaza Saeidi-Javash,[1,8] Hirotaka Sakaue,[1] Babak Anasori,[7] Lihua Jin,[3] Michael D. McMurtrey,[9] and Yanliang Zhang[1]✉

[1]Department of Aerospace and Mechanical Engineering, University of Notre Dame, Notre Dame, IN 46556, USA

[2]Department of Chemical Engineering, Texas Tech University, Lubbock, TX 79409, USA

[3]Department of Mechanical and Aerospace Engineering, University of California Los Angeles, Los Angeles, CA 90095, USA

[4]Department of Mechanical Science and Engineering, University of Illinois Urbana-Champaign, Urbana, IL 61801, USA

[5]Materials Measurement Laboratory, National Institute of Standards and Technology, Gaithersburg, MD 20899, USA

[6]Department of Materials Science and Engineering, University of Maryland, College Park, MD 20742, USA

[7]Department of Mechanical and Energy Engineering and Integrated Nanosystems Development Institute, Purdue School of Engineering and Technology, Indiana University−Purdue University Indianapolis, Indianapolis, Indiana 46202, USA

[8]Department of Mechanical and Aerospace Engineering, California State University Long Beach, Long Beach, CA 90840, USA

[9]Idaho National Laboratory, Idaho Falls, ID 83415, USA

[10]These authors contributed equally: Minxiang Zeng, Yipu Du, Qiang Jiang.

✉Corresponding Author: yzhang45@nd.edu.

Table of Contents

Supplemental Methods 4

Synthesis of 0D nanospheres 4

Synthesis of 1D nanowires 4

Synthesis of 2D nanoplates/nanosheets 4

Printing of functionally graded polyurethane films 5

Materials characterization 5

Fluorescent imaging of printed functionally graded polyurethane films 6

Seebeck coefficient and electrical conductivity measurement 6

Property mapping of printed gradient polyurethane films 7

Supplemental Figures 9

Fig. S1. Schematic demonstration of the gradient design and printed pattern........ 9

Fig. S2. Printing resolution and comparison of two printing strategies........ 10

Fig. S3. Printing optimization of ink flow rate (**a**), sheath flow (**b**), print speed (**c**), and atomizing voltage (**d**) with the use of $Sb_{1.5}Bi_{0.5}Te_3$ nanoplate ink.. 11

Fig. S4. The sheet resistance of printed thin films with different printing times........ 12

Fig. S5. The sample-to-sample consistency of printed thin films........ 13

Fig. S6. The batch-to-batch consistency of printed thin films over 6 months. Each condition was printed with four replicates to calculate the standard deviation. 14

Fig. S7. Deposition thickness (black) and relative uncertainty (red) through changing printing speed (**a**), printing layer (**b**), ink flow rate (**c**), and atomizing voltage (**d**). 15

Fig. S8. Correlation matrix of deposition thickness versus different printing parameters. 16

Fig. S9. The thickness profile of a gradient film of low-concentration ink. 17

Fig. S10. Reduced surface roughness by optimizing ink formulations. 18

Fig. S11. Consistency analysis of combinatorial printing using 13 gradient serpentine lines.. 19

Fig. S12. Relative uncertainty of combinatorial printing using 13 gradient serpentine lines... 20

Fig. S13. Demonstration of printing chalcogenide inks on various substrates........ 21

Fig. S14. Demonstration of printing graphene inks on various substrates 22

Fig. S15. Fast camera images of aerosol jet printing process with a sheath flow rate of 40 sccm (**a**), 30 sccm (**b**), 20 sccm (**c**), 10 sccm (**d**). 23

Fig. S16. Time-dependent fast camera images of the aerosol jet process. 24

Fig. S17. Aerosol streams of photoluminescent inks during printing........ 25

Fig. S18. Sample-to-sample reproducibility of 3 gradient Ag/Bi_2Te_3 samples (with balance Ag, Bi, and Te) printed under the same conditions. 26

Fig. S19. TEM (**a**) and EDS images of nanocomposites of Bi_2Te_3 and Ag particles with Te (**b**), Bi (**c**), and Ag mapping (**d**). 27

Fig. S20. CFD simulation model with geometry and boundary conditions 28

Fig. S21. CFD simulation on aerosol mixing and jetting in HTCP under sheath flows. 29

Fig. S22. Comparison of aerosol mixing with CFD simulation. 30

Fig. S23. CFD simulation on the velocity profile of low sheath flow condition (**a**) and high sheath flow condition (**b**). 31

Fig. S24. Effect of aerosol mixing and printing. 32

Fig. S25. Ink incompatibility by conventional mixing due to charge mismatch 33

Fig. S26. Comparison of HTCP with conventional ink mixing 34

Fig. S27. Elemental analyses of various type of combinatorial material libraries that contains elements from s-block, d-block, and p-block groups of the periodic table.. 35

Fig. S28. SEM image of a cross-sectional view of nanocomposite of Bi_2Te_3 and Ag particles. 36

Fig. S29. Combinatorial sulfur doping of n-type $Bi_2Te_{2.7}Se_{0.3}$.. 37

Fig. S30. The thermoelectric power factor of extrusion printed $Bi_2Te_{2.7}Se_{0.3}$ samples with various sulfur doping concentrations. 38

Fig. S31. Combinatorial doping/alloying for active tuning of the transport behaviors of charge carriers.. 39

Fig. S32. Young's modulus of functionally graded polyurethane films in comparison with that of biomaterials[16]. 40

Fig. S33. Young's modulus of functionally graded polyurethane films vs sample locations measured during 4 repeated stretching-releasing cycles. 41

Fig. S34. Combinatorial reaction of GO with reducing agent. 42

Supplemental Tables 43

Table S1: Comparison of typical processing parameters of different combinatorial fabrication methods. 43

Table S2. Typical ink formulation of nanoparticle inks. 44

Table S3. Aerosol jet printing parameters of combinatorial thermoelectric films. 45

Table S4. Aerosol jet printing parameters of graphene films 46

Table S5. Aerosol jet printing of graphene films with different printing speeds 47

Table S6. Parameter table of CFD simulation 48

Table S7. Comparison of printed thermoelectric materials on their thermoelectric power factors 49

Supplementary References 50

Supplemental Methods

Synthesis of 0D nanospheres

Spherical polystyrene nanospheres were synthesized by an emulsion polymerization method[1]. In a typical experiment, 19.0 g styrene, 1.0 g methyl methacrylate, and 1.0 g acrylic acid were added into 200 mL H_2O in a 500 mL glass flask. The mixture was first stirred for 10 minutes under N_2, followed by heating to 80 °C. Then, 0.483 g ammonium persulfate, 2 mg-6 mg sodium dodecylbenzenesulfonate, and 500 mg $NaHCO_3$ were added. After mixing homogeneously, the above mixture was continuously stirred at 80 °C for 7 hours. Monodispersed polystyrene colloidal nanoparticles were further purified by washing with deionized (DI) water three times.

Synthesis of 1D nanowires

2.7 mmol Na_2TeO_3 and 1.5 g NaOH were dissolved in 70 mL ethylene glycol (EG). Then, 0.5 g polyvinylpyrrolidone (PVP) was added. The mixture solution was refluxed at 180 °C for 6 hours under Ar protection. Once the system was cooled to room temperature, a small amount of acetone was added to precipitate the Te nanowires which were then cleaned and redispersed by ethanol. The cleaning steps were repeated three times to remove any excess PVP and unreacted starting materials.

Synthesis of 2D nanoplates/nanosheets

The 2D nanoplates/nanosheets were prepared via either bottom-up colloidal synthesis or top-down exfoliation approach. For example, to synthesize few-layer graphene inks, 2 g expanded graphite (Asbury Carbons, grade 3805) was introduced to a surfactant solution (graphene quantum dots[2]) followed by the tip-sonication for 1 h. To eliminate unexfoliated bulk crystals, the dispersion was subjected to a centrifugation process at 2000 rpm for a duration of 30 minutes. The graphene nanosheets were further purified by removing excess ions using ion exchange resins prior to ink formulation. For graphene oxide (GO) inks, the graphene oxide nanosheets are prepared by a modified Hummers' method. In a typical synthesis, the graphite was first treated by microwave irradiation for 90 s (1000 W) to obtain expanded graphite. The expanded graphite powders (5 g) were mixed with a solution of concentrated H_2SO_4 (115 mL) and H_3PO_4

(25 mL) in an ice bath. $KMnO_4$ (20 g) was then carefully added to the mixture and allowed to react for 4 h, followed by the gradual addition of DI water (230 mL). The addition of DI water (300 mL) containing hydrogen peroxide (3 wt.%, 50 mL) followed. After centrifugation, the solid obtained was washed several times with excess DI water, concentrated hydrochloric acid, and DI water, until the pH of the solution reached near neutral. This final aqueous solution of GO sheets was then stored in the refrigerator before printing. For MXene nanosheets ($Ti_3C_2T_x$), we synthesized the 2D flakes by selective etching of aluminum from its respective MXene precursor, Ti_3AlC_2, using a combined hydrofluoric acid-hydrochloric acid (HF-HCl) mixture and lithium chloride for delamination, as described elsewhere[3,4].

Printing of functionally graded polyurethane films

Polyurethane dispersions (PUD) U 9380 (high modulus) was bought from Alberdingk Boley and diluted with water and ethylene glycol (PUD: water: ethylene glycol = 6:11:5). Polyurethane dispersions CD102 (low modulus) was bought from Baymedix and diluted with water, dimethylformamide (DMF), and ethylene glycol (PUD: water: DMF: ethylene glycol = 6:7:4:3). Ink flow rates of the two inks are programmed ranging from 9 to 20 sccm, while the sheath flow rate is 60 sccm and the atomizer voltage is 38V.

Materials characterization

SEM images of the samples were obtained using a focused ion beam-scanning electron microscope (FIB-SEM, Helios G4 UX). A transmission electron microscope (TEM, Titan 80-300) was used to image the TEM samples. A confocal Raman microscope (NRS-5100, Jasco) with high-Speed XYZ imaging capability was used to characterize material structure and composition (*e.g.*, combinatorial polymers). To map the elemental distribution of combinatorial films, energy-dispersive X-ray spectroscopy (Helios G4 UX and Titan 80-300) and X-ray fluorescence imaging (EDAX Orbis PC Micro-XRF) were used depending on the sample compositions and dimensions. A high-speed color camera (MEMRECAM HX-7s) was used to investigate the aerosol stream during printing. A UV head and a driver (SOLIS-365C, DC-20) were used to excite the aerosol stream of photoluminescent inks. A digital single-lens reflex (SLR) camera (EOS Kiss

X5) captured images of the luminescent output from the excited aerosol stream. An optical long-pass filter (ZVL0430) was placed in front of the camera and cut off shorter than 430 nm wavelength.

Fluorescent imaging of printed functionally graded polyurethane films

To investigate the gradient composition of printed polyurethane films, we mixed two PUD inks with different fluorescent dyes, followed by fluorescent imaging. Specifically, a red color dye and a green color dye were added to the PUDs to better visualize the composition change process. The fluorescent dyes were purchased from GLO EFFEX with red (UVT-RD-1OZ) and green (UVT-GR-1OZ). The fluorescent photographs were captured through Keyence BZ-X810 Microscope. To quantify the intensity of the two colors, we did RGB analyses on the printed gradient polyurethane films, where the RGB color profiles were obtained by using the ImageJ RGB-Profiler plugin (National Institutes of Health, USA).

Seebeck coefficient and electrical conductivity measurement

The printed gradient combinatorial films are characterized using either a custom-built probing system at the University of Notre Dame or a custom-built scanning thermoelectric instrument at the National Institute of Standards and Technology (NIST). In our custom-built probing system at the University of Notre Dame, two fine 40 AWG k-type thermocouples were placed ~1 mm apart and a heater was placed ~1 mm away from one of the thermocouples. Two electrodes through which current is sent for electrical conductivity measurement are placed at both ends of the film. At each measurement location, electrical conductivity is measured when the film is at thermal equilibrium with the surroundings before measuring the Seebeck coefficient. During the Seebeck coefficient measurement, the heating power is slowly increased and the Seebeck voltage between the two thermocouples is collected continuously along with the thermocouple temperatures. After measurement, the negative of slope of the best fit line of the Seebeck voltage versus the temperature difference between the thermocouples gives the measured Seebeck coefficient relative to the thermocouple wire with which the Seebeck voltage was measured. The absolute Seebeck coefficient of the film is the sum of the measured Seebeck coefficient and the Seebeck coefficient of the thermocouple wire. The probe was scanned along the length of the film with a distance between measurement locations

equal to 1/10th the thermocouple spacing. A custom-built high-throughput scanning instrument was used to measure the Seebeck coefficient of gradient Sb-Bi-Te samples at NIST[5]. The Seebeck coefficient was measured in 0.5 mm increments using a pair of independently spring-loaded Type R thermocouple probes spaced 3 mm apart, using the quasi-steady state condition of the differential method[6]. A small rising temperature difference was applied to the film using a heater element embedded in one of the thermocouple probes. The thermoelectric voltage and temperature differences were each recorded at five intervals between 1 K and 5 K. The slope of the unconstrained linear fit of the voltage and temperature difference ordered pairs ($R^2 \geq 0.95$) was calculated as the Seebeck coefficient. Since the voltage and the hot and cold thermocouples were measured in sequence, the Seebeck coefficient obtained using one sequence and its inverse were averaged to correct correspondence distortion errors[6]. The measured Seebeck coefficient was then subtracted from the absolute Seebeck coefficient of the platinum reference wires to calculate the corrected Seebeck coefficient of the film. The measurement uncertainty (one standard deviation) for the Seebeck measurement is ± 6.5 %.

In addition, the measurements were validated by measuring the same bulk constantan film using a well-established bulk thermoelectric measurement.

Property mapping of printed gradient polyurethane films

We measured the Eulerian strain $\boldsymbol{e}$ by Ncorr[7], an open-source 2D DIC Matlab software, in the deformed state. The Cartesian coordinate is chosen such that the length and stretching of the sample are along the *y* direction, and the width is along the *x* direction. The Eulerian strain $\boldsymbol{e}$ relates to the left Cauchy-Green tensor $\mathbf{B}$ by $\mathbf{B} = (\boldsymbol{I} - 2\boldsymbol{e})^{-1}$. Finding the eigenvalues and eigenvectors of $\mathbf{B}$ as λ_i^2 and $\mathbf{v}_i$ (*i*=1-3), respectively, we could obtain the logarithmic strain in the principal direction as

$$\hat{\boldsymbol{\varepsilon}} = \sum_{i=1}^{3} \ln(\lambda_i)\mathbf{v}_i \otimes \mathbf{v}_i, \qquad (1)$$

and transform it back to the *x-y* Cartesian coordinate as

$$\boldsymbol{\varepsilon} = \boldsymbol{V} * \hat{\boldsymbol{\varepsilon}} * \boldsymbol{V}^{-1}, \quad (2)$$

where $\boldsymbol{V} = [\mathbf{v}_1, \mathbf{v}_2, \mathbf{v}_3]$. Similarly, we can calculate the engineering strain in the principal direction as $\hat{\boldsymbol{\varepsilon}}^{\text{eng}}$ and the *x-y* Cartesian coordinate as $\boldsymbol{\varepsilon}^{\text{eng}}$

$$\hat{\boldsymbol{\varepsilon}}^{\text{eng}} = \sum_{i=1}^{3} \lambda_i \mathbf{v}_i \otimes \mathbf{v}_i, \quad (3)$$

$$\boldsymbol{\varepsilon}^{\text{eng}} = \boldsymbol{V} * \hat{\boldsymbol{\varepsilon}}^{\text{eng}} * \boldsymbol{V}^{-1}. \quad (4)$$

The true stress was then calculated as

$$\sigma_{yy} = \frac{F}{a} = \frac{F}{A\left(1+\varepsilon_{xx}^{eng}\right)^2}, \quad (5)$$

where F is the loading force, A and a are the cross-section area before and after deformation, respectively, and ε_{xx}^{eng} is the *xx* component of $\boldsymbol{\varepsilon}^{\text{eng}}$ obtained in Eq. 4. Assuming the material is linearly elastic, and σ_{xx} and σ_{zz} are negligible, we obtained the distribution of Young's modulus in the deformed configuration $E(y)$

$$E(y) = \frac{\sigma_{yy}}{\varepsilon_{yy}(y)}, \quad (6)$$

where ε_{yy} is the *yy* component of $\boldsymbol{\varepsilon}$ obtained in Eq. 2. $E(y)$ was further converted to the modulus distribution as a function of *Y*, the coordinate along the stretching direction in the undeformed configuration, $E(Y)$, where $Y = y + V$ with V the displacement along the stretching direction obtained by Ncorr, and plotted in Fig. 4f.

Supplemental Figures

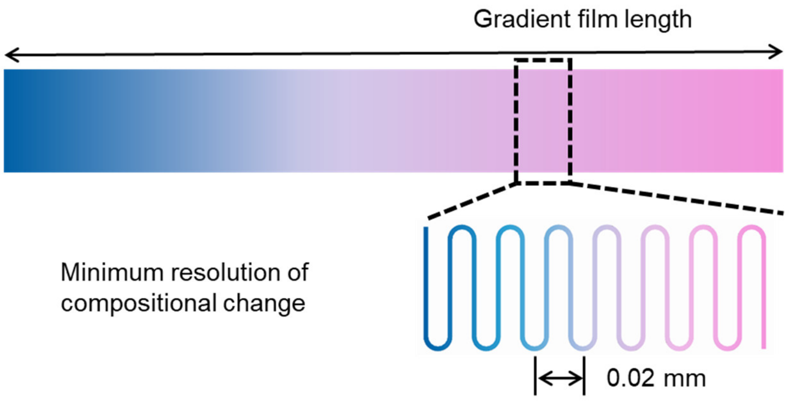

Fig. S1. Schematic demonstration of the gradient design and printed pattern. A design of gradient film consisting of individually connected printing pass that changes the deposition composition on the fly. A typical line gap is 0.02 mm.

To form a high-feature density of the gradient film, we set the composition change every 0.02 mm (Fig. S1). Given the fact that the deposited inks remain wet for a few seconds after printing, the consequent diffusion occurs among the adjacent composition in the ink phase, which leads to the formation of gradient thin films with compositionally varying features (Fig. 1).

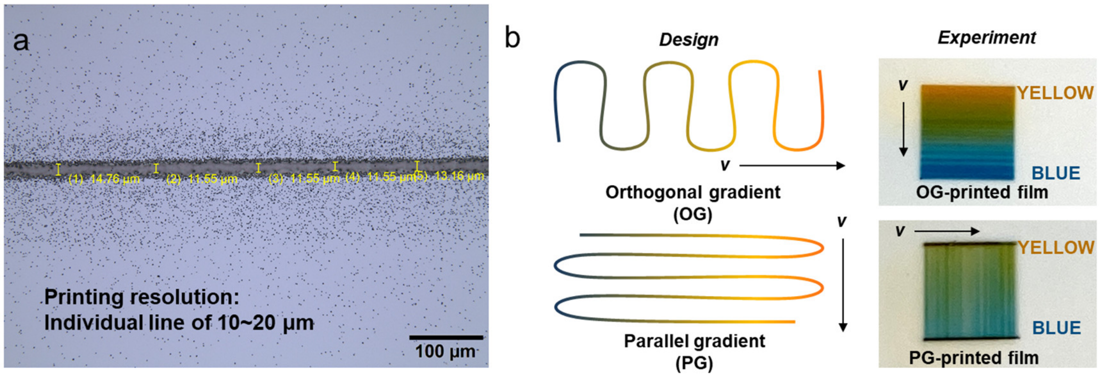

Fig. S2. Printing resolution and comparison of two printing strategies. **a**, Printing of an individual line showing the deposition resolution as small as 20 µm (Ink particle: Ag; nozzle size: 30 gauge). **b**, Design and experiment of orthogonal gradient (OG) and parallel gradient (PG) printing.

In a typical orthogonal gradient printing process, the printhead generates a uniform composition along the vertical direction, while it gradually changes the mixing ratio of two ink flow rates along the horizontal direction to enable the formation of a gradient film. By contrast, parallel gradient printing continually varies the ratio of the two ink flow rates along the printing direction when the printhead travels horizontally.

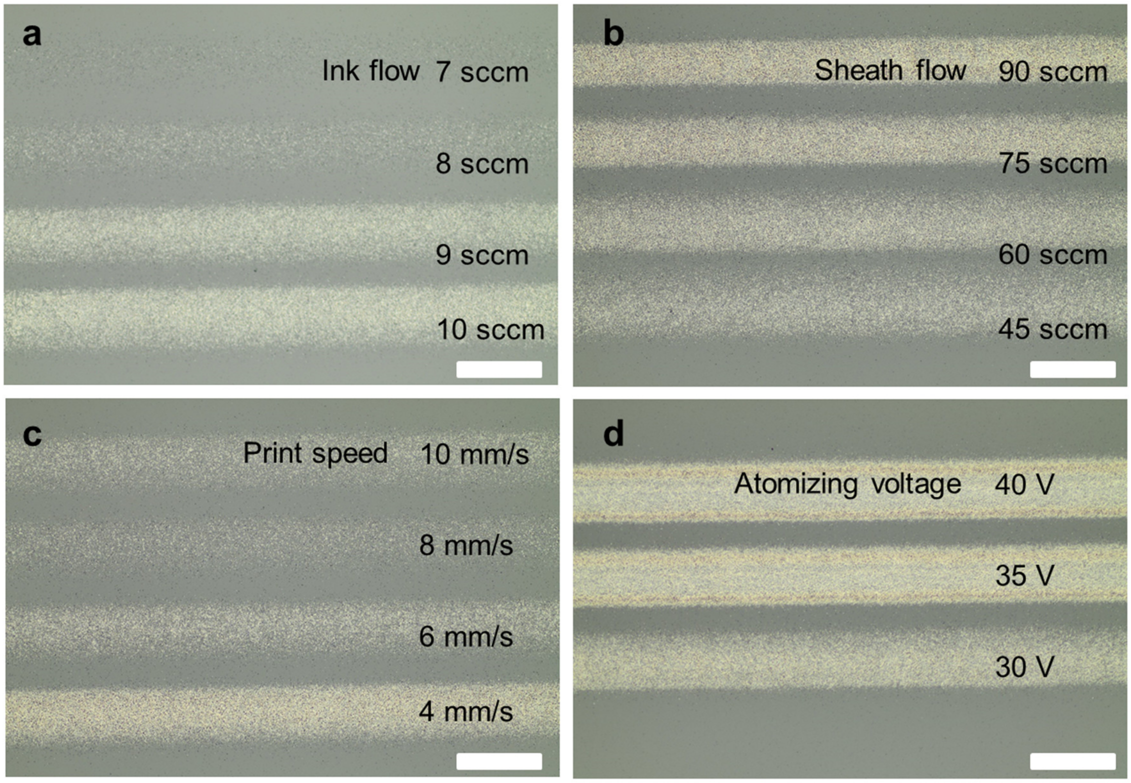

Fig. S3. Printing optimization of ink flow rate (**a**), sheath flow (**b**), print speed (**c**), and atomizing voltage (**d**) with the use of $Sb_{1.5}Bi_{0.5}Te_3$ nanoplate ink. Scale bar: 100 µm. Ink formation: 8 wt.% $Sb_{1.5}Bi_{0.5}Te_3$ nanoplates in water (15 wt.% ethylene glycol was added as co-solvent); nozzle size: 22 gauge (413 µm).

The optical microscopy images revealed that the amount of deposited nanoparticles increases monotonically with ink flow rate (7 sccm to 10 sccm), while an increase in print speed (4 mm/s to 10 mm/s) leads to less particle deposition per print pass. Under our experimental conditions (45 sccm to 90 sccm), a higher sheath flow can result in a narrower print line due to a stronger aerodynamic focusing. For $Sb_{1.5}Bi_{0.5}Te_3$ nanoplate ink, the deposited material will increase (30 V to 35 V) and then reach a plateau (35 V to 40 V) upon increasing the atomizing voltage.

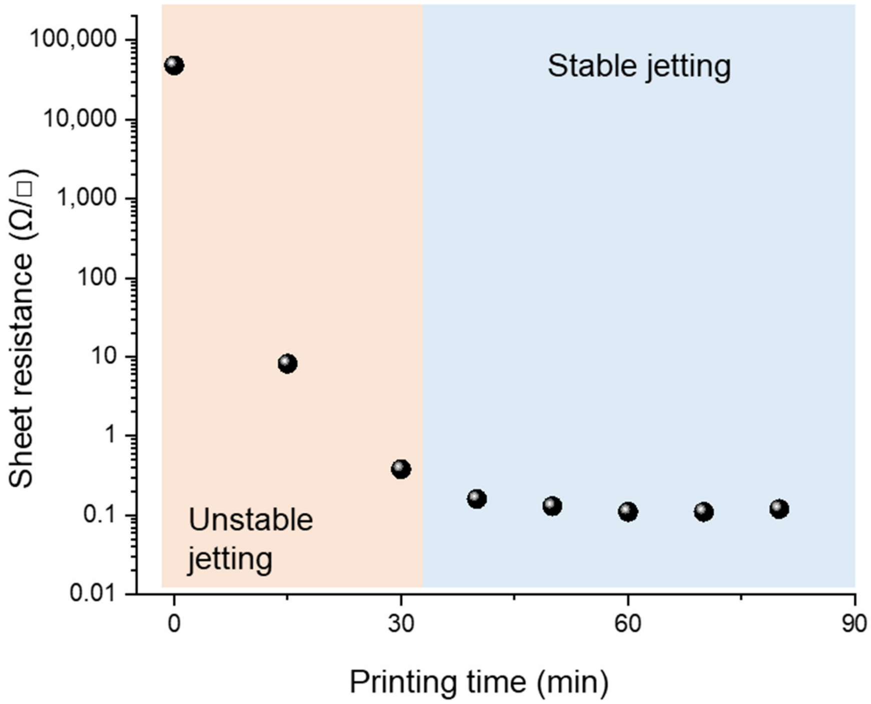

Fig. S4. The sheet resistance of printed thin films with different printing times. It reveals the inconsistent printing during the beginning of the printing (first 30 min), while the printing becomes steady due to the stable jetting and thus the sheet resistance is reproducible. Ink formation: 10 wt.% Au ink in xylene.

To understand the ink printing stability, the time-dependent printing of inks was studied. The aerosol jet printing requires a short period of time (*e.g.*, 30 min for 10 wt.% Au ink) as ink saturation time because 1) it allows ultrasonic atomizing stabilization; 2) it saturates the surface of flow tubes as aerosols pass through. This is seen in the time-dependent resistance measurement (Fig. S4), as the printed thin resistance changes dramatically in the first 30 min, and then it becomes steady due to stable jetting. The uncertainty comes from aerosol losses during transportation. In practice, longer transportation tubing requires greater ink flow rates for equivalent losses. In addition, maintaining the colloidal stability and chemical stability of the inks throughout the entire printing process is imperative to the printing consistency. For example, a steady aerosol jet printing requires the aerodynamic focusing of aerosol particles which typically excludes the use of highly volatile solvents (such as acetone, hexane, or dichloromethane), and thus solvents with relatively low vapor pressure are often preferred. With judicious ink engineering (solvent and additives), we enhanced the printing stability (Fig. S5) and shelf life of ink materials for over 6 months (Fig. S6).

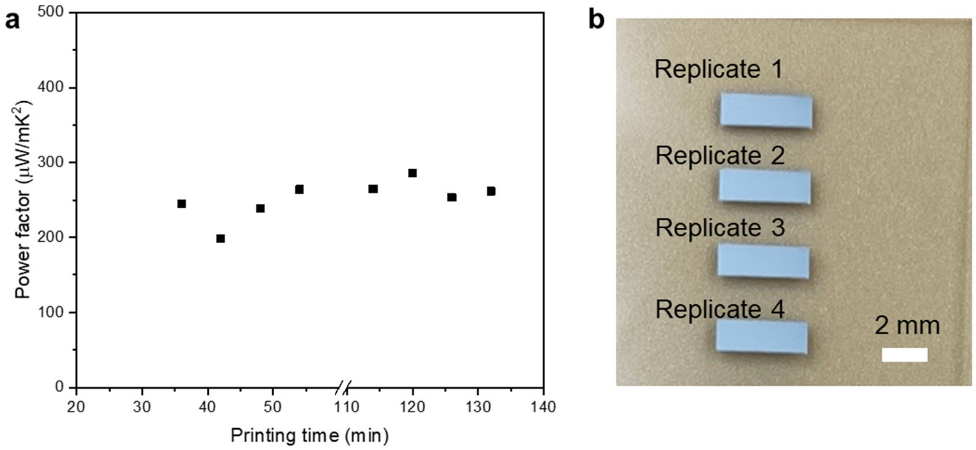

Fig. S5. The sample-to-sample consistency of printed thin films. **a**, Thermoelectric power factor of n-type bismuth telluride with different printing times. **b**, The photographic image of four replicates of printed thin films.

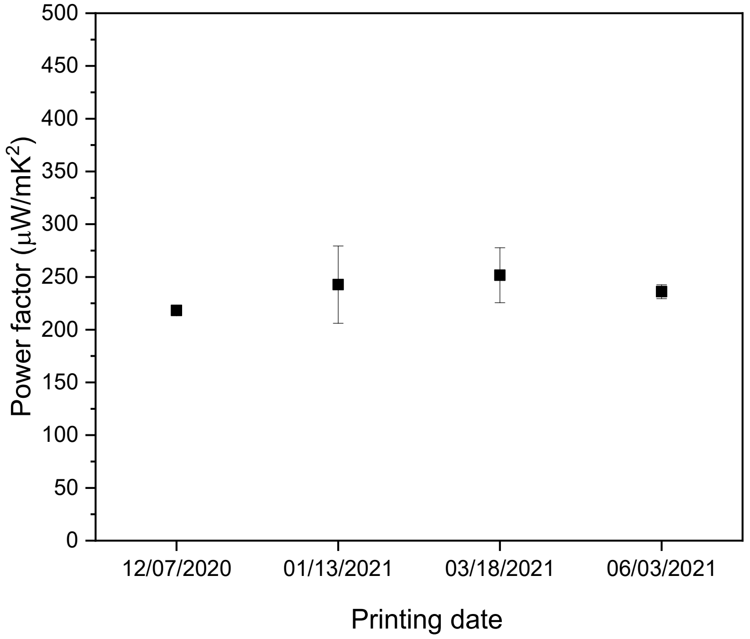

Fig. S6. The batch-to-batch consistency of printed thin films over 6 months. Each condition was printed with four replicates to calculate the standard deviation.

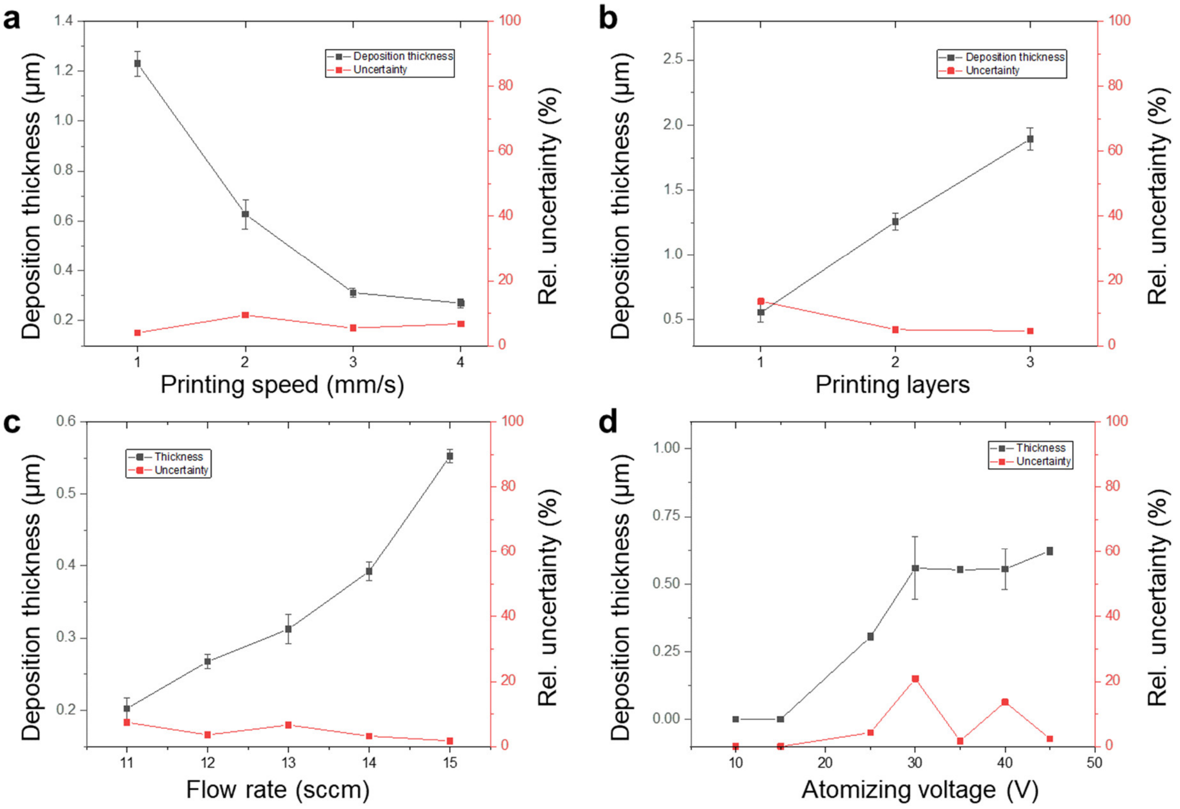

Fig. S7. Deposition thickness (black) and relative uncertainty (red) through changing printing speed (**a**), printing layer (**b**), ink flow rate (**c**), and atomizing voltage (**d**). Each condition was printed with four replicates to calculate the standard deviation.

We conducted a statistical analysis of the uncertainty. The aerosol-based printing involves multiple processing parameters. In order to reliably control the material deposition rate, careful control of the printing parameters is needed. Specifically, we investigated how different parameters influence the control of aerosol deposition. After priming the ink with enough time, the printed films showed a high correlation between the flow rate and deposition thickness. In addition, we test the effect of printing speed, printing layer, and atomizing voltage, which exclusively showed reasonable uncertainty (<20%). The highest uncertainty comes from the low-to-medium atomizing voltage which is likely due to the inefficient atomizing (atomizing voltage ≤ 30 V).

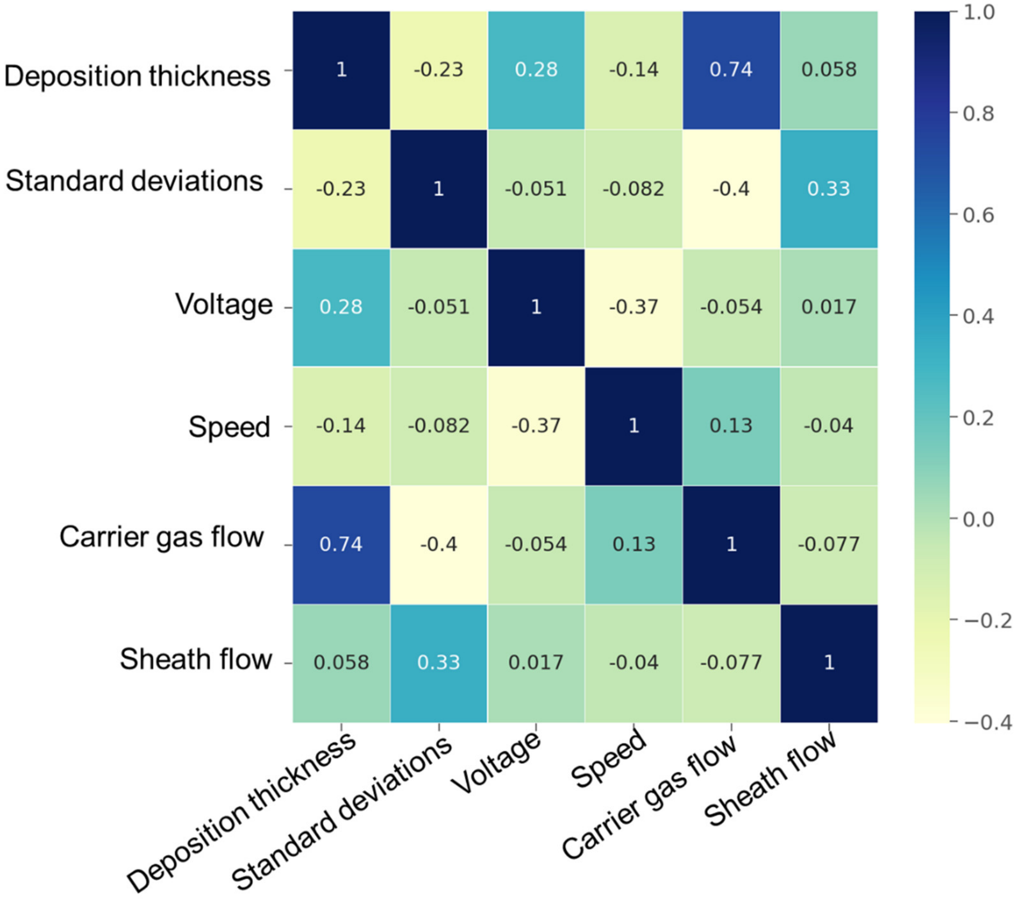

Fig. S8. Correlation matrix of deposition thickness versus different printing parameters.

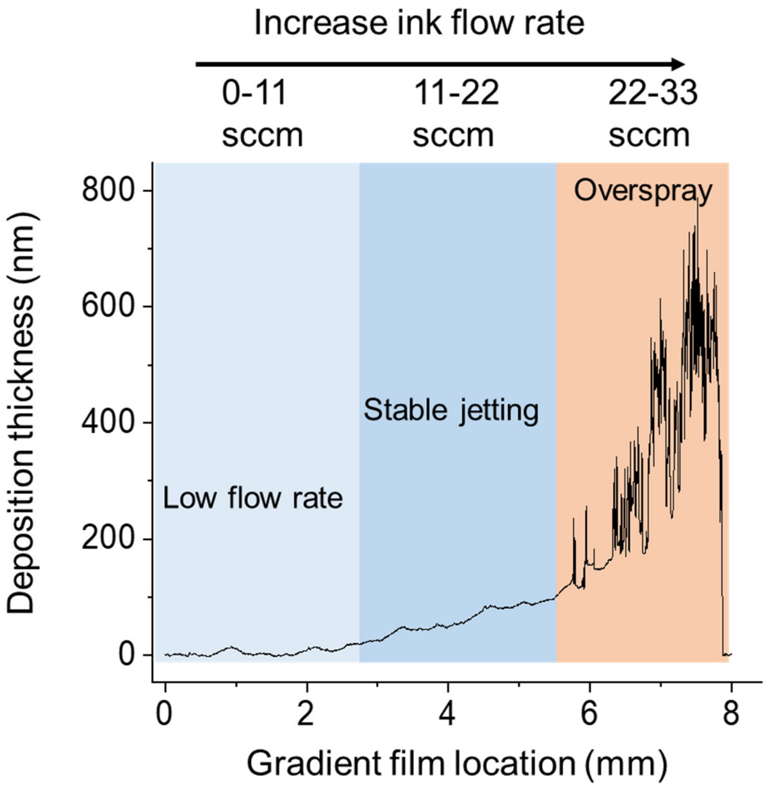

Fig. S9. The thickness profile of a gradient film of low-concentration ink (graphene ink) when increasing ink flow rate from 0 sccm to 33 sccm, which reveals three distinctive printing regions: low flow rate region, stable jetting, and overspray region. Ink concentration: 0.2 wt%, sheath flow: 60 sccm, print speed: 2 mm/s.

For an aqueous graphene ink (0.2 wt%), we gradually increased the ink flow rate from 0 sccm to 33 sccm and identified three distinctive printing regions: low flow rate region, stable jetting, and overspray/unstable jetting with high uncertainty (shown in the above figure).

For our combinatorial printing, we focus on the stable jetting region by calibrating the individual line. An increase in print passes or a decrease in print speed can further reduce the deposition uncertainty. It is also worth noting that the above flow range may vary depending on the type of ink particle, solid content, and solvents. A change in sheath flow could also influence the optimized printing range and overspray.

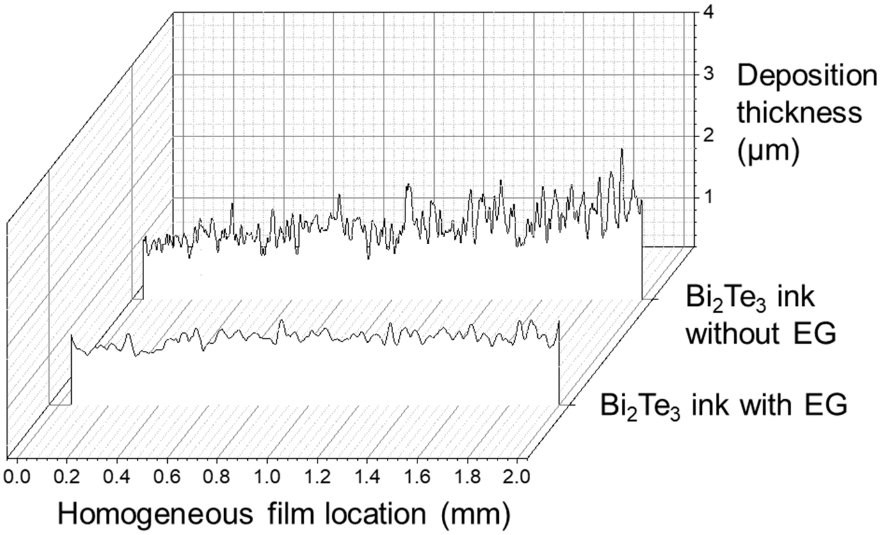

Fig. S10. Reduced surface roughness by optimizing ink formulations. Ink formation: 6 wt.% Bi_2Te_3 in water and water/EG inks (2 print passes).

The ink formulation is important to printing optimization. It was found that the addition of the high-boiling-point solvent ethylene glycol (EG) can alleviate the fast-drying effect of the printed nanomaterial inks, leading to a smoother surface and better controllability in deposition thickness (The detailed ink information can be found in the Supplemental Tables). In addition, the high boiling point of EG in inks can also affect aerosol deposition in a way that the average size of these aerosols tends to be bigger than that of aerosols made from pure water due to the reduced evaporation effect. During such processes, larger aerosol droplets experience a stronger Saffman force as the Saffman force is proportional to the square of particle size[8]. This helps to narrow the passage of the aerosol flow and improve the printing performance. Additional discussion on the relationship between aerosol size, Saffman force, and aerosol printing can be found in the fast camera section (Fig. S15).

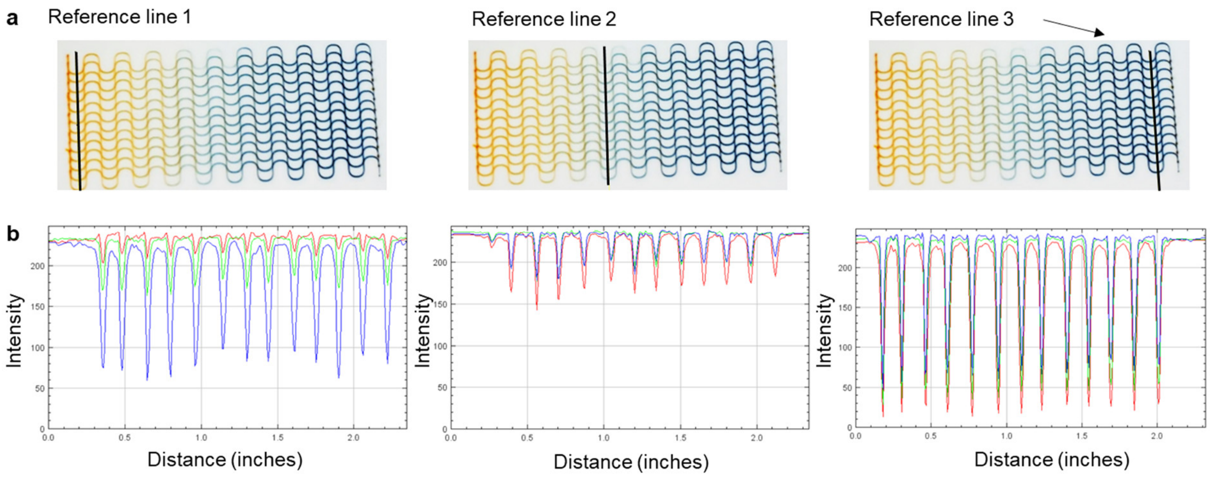

Fig. S11. Consistency analysis of combinatorial printing using 13 gradient serpentine lines. **a**, Print of 13 serpentine lines of gradient mixing of food dye inks showing almost identical color distribution behaviors. **b**, RGB profile of 13 printed gradient serpentine lines at three different reference points, showing reasonable repeatability.

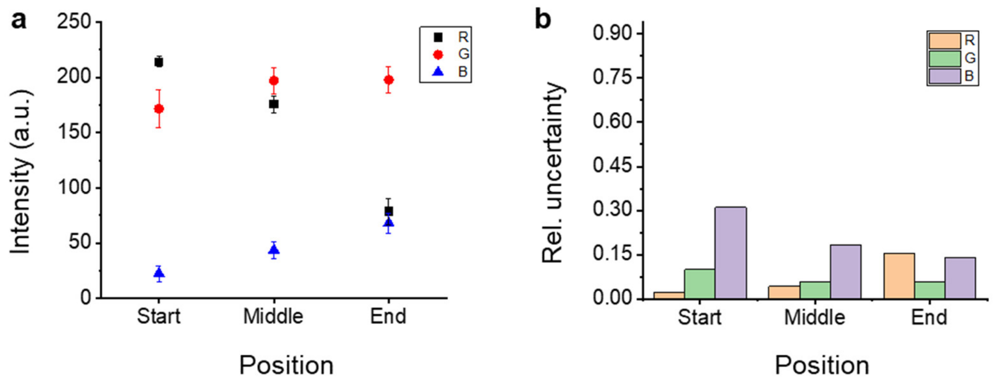

Fig. S12. Relative uncertainty of combinatorial printing using 13 gradient serpentine lines. **a**, Average RGB values of 13 serpentine lines at starting, middle, and ending points. Error bars represent standard deviations from 13 experimental replicates. **b**, The relative uncertainty of RGB values of 13 serpentine lines at starting, middle, and ending points. The relative uncertainty is calculated as the sample standard deviation divided by the average value.

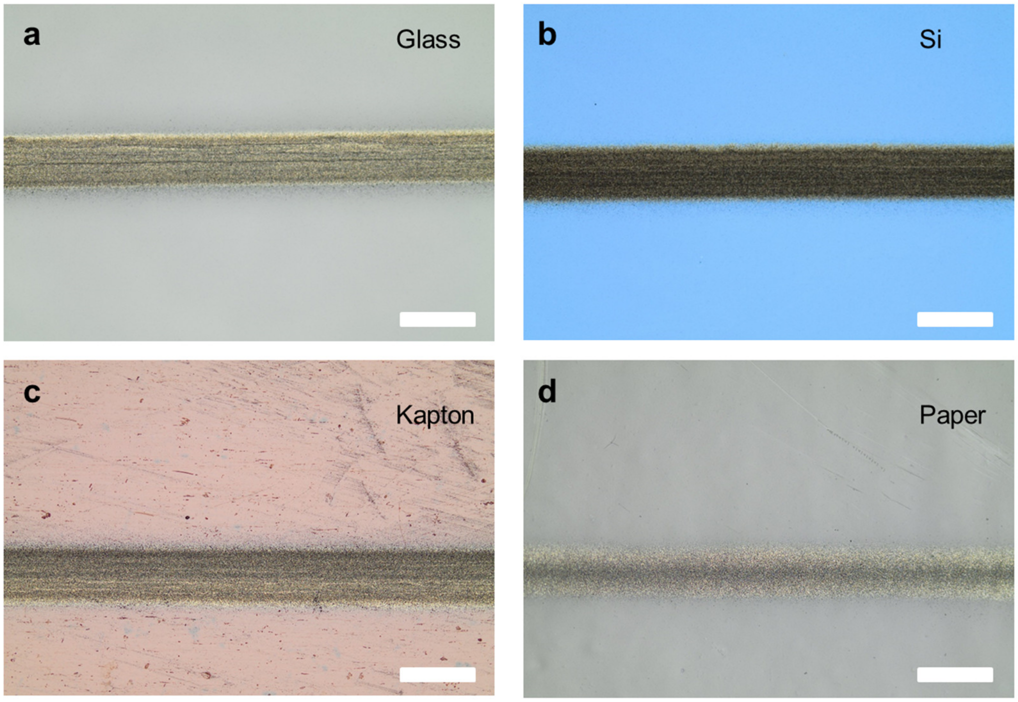

Fig. S13. Demonstration of printing chalcogenide inks on various substrates including glass (**a**), Si (**b**), Kapton (**c**), and paper (**d**). Scale bar: 100 µm. Nozzle size: 22 gauge (413 µm).

The HTCP method is relatively insensitive to the substrates. The chalcogenide particle ink has been successfully printed on various types of substrates.

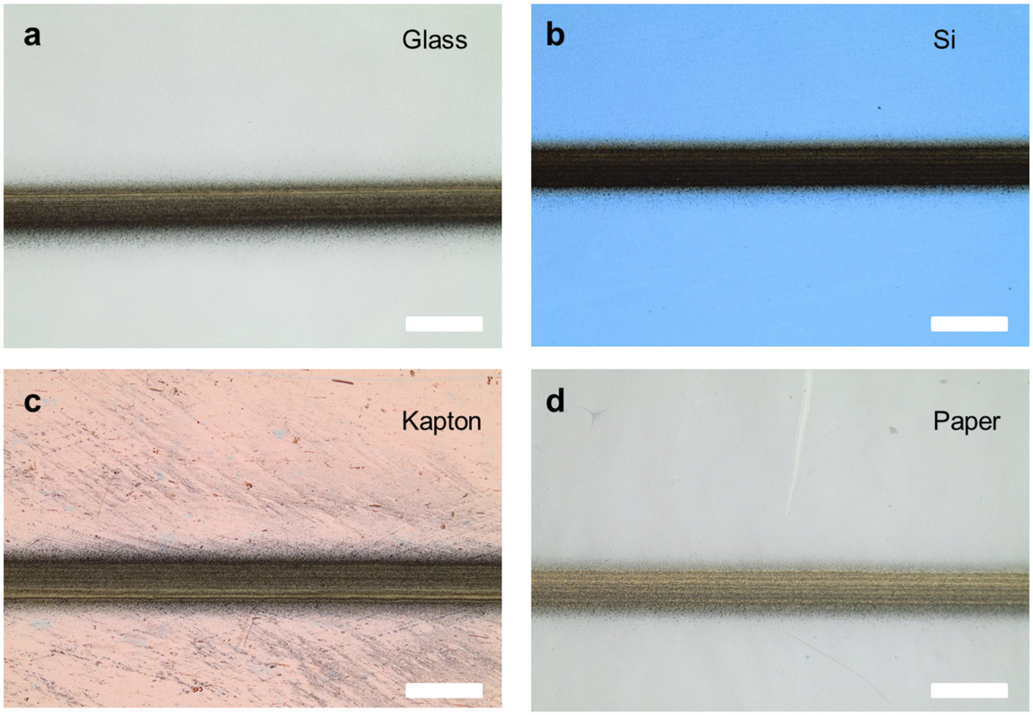

Fig. S14. Demonstration of printing graphene inks on various substrates including glass (**a**), Si (**b**), Kapton (**c**), and paper (**d**). Scale bar: 100 µm. Nozzle size: 22 gauge (413 µm).

We also evaluated the substrate effect on another ink (graphene ink) via the HTCP method. Similar to chalcogenide particle ink, graphene inks can be successfully printed on various substrates including glass, Si, Kapton, and paper.

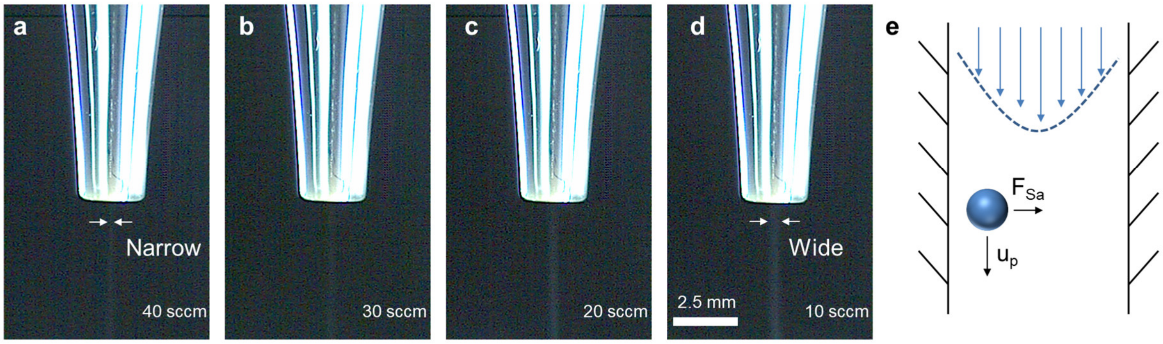

Fig. S15. Fast camera images of aerosol jet printing process with a sheath flow rate of 40 sccm (**a**), 30 sccm (**b**), 20 sccm (**c**), 10 sccm (**d**). To facilitate the imaging of the aerosol stream, a custom-built glass nozzle was used (internal diameter of ~1.70 mm). **e**, Schematic demonstration of Saffman force (F_{Sa}) on aerosol droplets with a velocity of u_p.

Upon decreasing the sheath flow rate from 40 sccm to 10 sccm, it was observed that the aerosol stream became increasingly wide. This indicates the significant role of sheath flow in aerodynamic focusing during HTCP. The high-speed camera videos can be found in Supplementary Video S2. The enhanced sheath flow rate has a strong collimating action and narrows the passage of the ink aerosols, which is reminiscent of the observation of blood corpuscles in the capillaries that tend to avoid vessel walls[9].

To understand the role of sheath flow in determining the flow behavior of small aerosol droplets in printing nozzles, we consider an aerosol droplet as a near-spherical particle (as shown in the above figure), which is transported within the Poiseuille flow at a velocity of u_p. Following the pioneering work by Akhatov et al. [10], it was suggested that the droplet not only experiences particle inertia and the Stokes force from gas–particle interaction, but also the Saffman force acting on aerosol droplets in gas flowing through a narrow nozzle thus triggering considerable migration of particles toward the centerline of the capillary. During such processes, larger aerosol droplets experience a larger Saffman force since the Saffman force is proportional to the square of particle size[8]. In addition, a higher sheath flow rate increases the relative velocity and shear rate in the z direction, which can lead to an enhanced Saffman force that helps to narrow the passage of the aerosol flow.

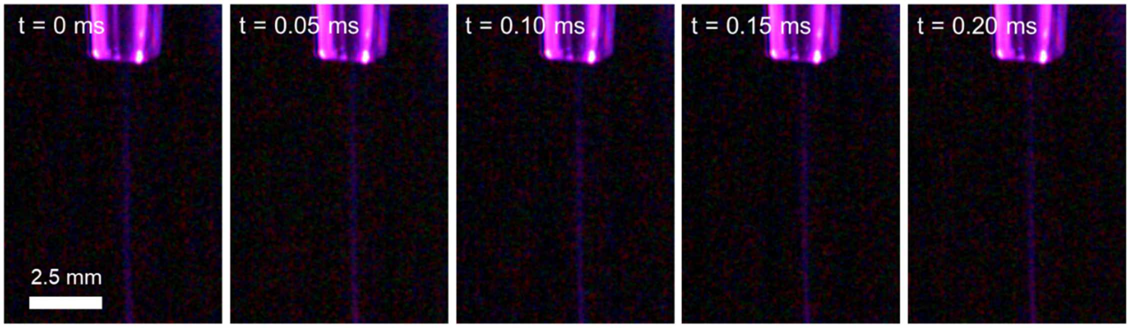

Fig. S16. Time-dependent fast camera images of the aerosol jet process.

By using fluorescent dyes as inks, we were able to image the aerosol stream using a high-speed camera (frame rate: 20000/s). To image the aerosol stream, we used a high-speed color camera system (MEMRECAM HX-7s). The color camera captured the fluorescence of the luminescent dyes, while a UV LED system excited the fluorescent dyes of ink aerosols. Undesired light was cut off using an optical long-pass filter which was placed in front of the camera. The collimated aerosol beam tends to be a straight stream while the focal region is not significantly widened, which is in direct contrast to low divergence aerosol particle beams from thin plate orifice nozzles or abruptly converging nozzles [11,12].

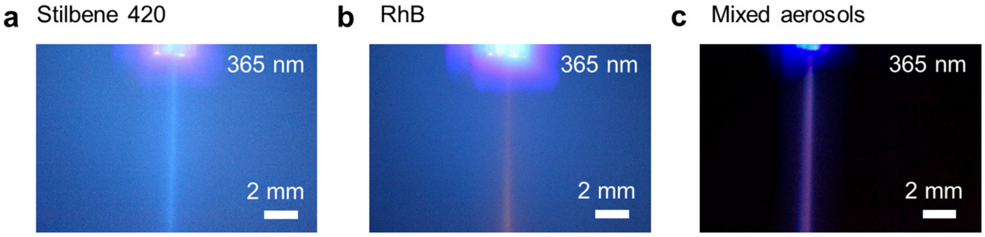

Fig. S17. Aerosol streams of photoluminescent inks during printing. **a**, Fast camera image of blue ink aerosols. **b**, Fast camera image of red ink aerosols. **c**, Fast camera image of HTCP mixed aerosols of blue and red inks.

To investigate the mixing behavior of HTCP, we used two fluorescent dyes as inks where stilbene 420 served as a blue dye and rhodamine B served as a red dye. A UV head and a driver (SOLIS-365C, DC-20) were used to excite the aerosol stream of photoluminescent inks. A digital single-lens reflex (SLR) camera (EOS Kiss X5) captured images of the luminescent output from the excited aerosol stream. As shown in the above figure, we observed a blue aerosol stream when jetting the ink of stilbene 420, while a red stream of aerosol was seen when we switched to rhodamine B ink (RhB). In addition, we observed the mixing of two aerosol jets by adjusting the printing parameters using two aerosol streams.

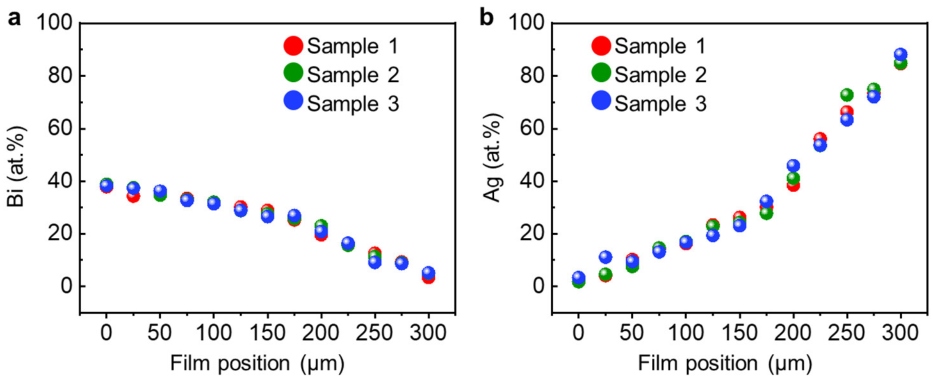

Fig. S18. Sample-to-sample reproducibility of 3 gradient Ag/Bi_2Te_3 samples (with balance Ag, Bi, and Te) printed under the same conditions. **a**, Bi content vs. film position among 3 gradient samples. **b**, Ag content vs. film position among 3 gradient samples.

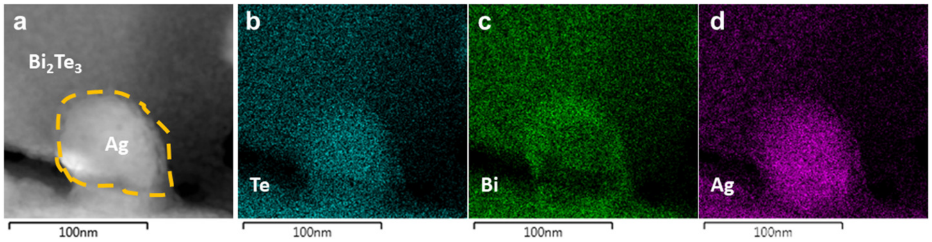

Fig. S19. TEM (**a**) and EDS images of nanocomposites of Bi_2Te_3 and Ag particles with Te (**b**), Bi (**c**), and Ag mapping (**d**).

The TEM image shows a silver nanoparticle grafted on Bi_2Te_3 nanoplates, while the EDS images reveal that the mixture of Ag and Bi_2Te_3 after thermal annealing can form a chemically blended composite in direct contrast to merely physical interaction. As shown in the above figure, we observed the diffusion of Te and Bi atoms into Ag nanoparticles as well as the existence of Ag atoms in the Bi_2Te_3 nanoplates, indicating the formation of ternary metal telluride. It is also interesting to observe that there are more Te, compared with Bi (see the empty region of Bi mapping), diffused into the Ag nanoparticles, which indicates a favored formation of silver telluride.

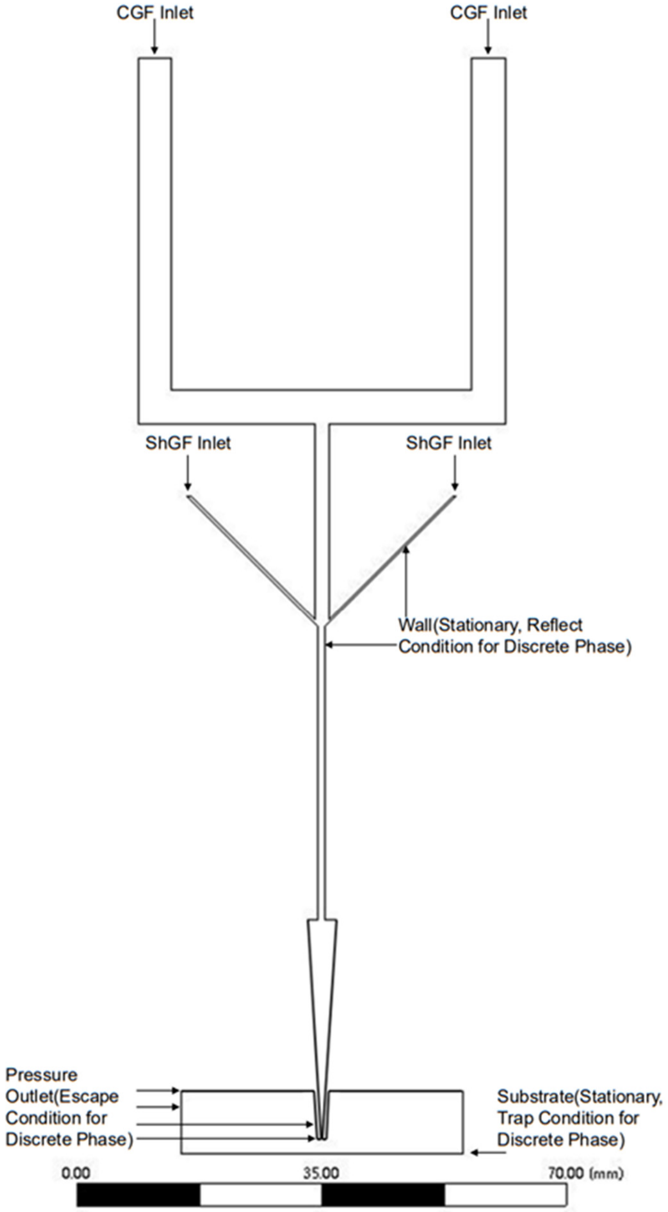

Fig. S20. CFD simulation model with geometry and boundary conditions.

In the simulation, velocity-inlet was set for both carrier gas flow (CGF, which is also known as ink flow) and sheath gas flow (ShGF) inlet. Stationary-wall condition, no-slip condition, and reflect condition were chosen for the interior surface wall. All the simulations were conducted using ANSYS FLUENT.

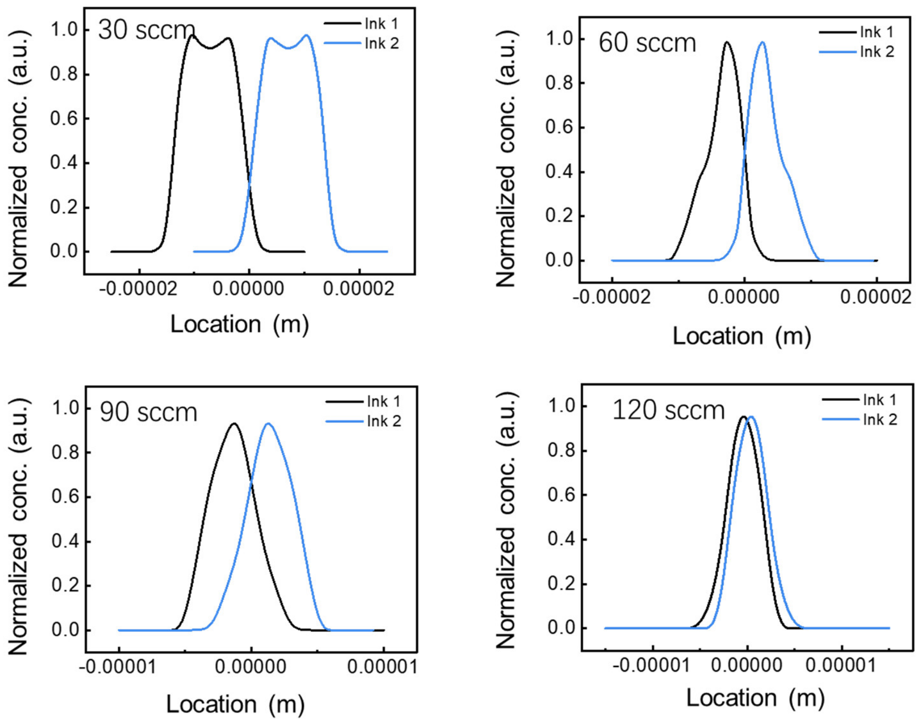

Fig. S21. CFD simulation on aerosol mixing and jetting in HTCP under sheath flows. It shows that the sheath flow can significantly influence the concentration profile of ink aerosols. A higher sheath can contribute to narrowing the passage of the ink gas flow, leading to a higher overlap of two inks.

To evaluate the mixing behavior under different sheath flows, we consider the scenario of equal CGF, where two discrete phases (*i.e.*, aerosols) are injected at the same flow velocity (see simulation details in Table S6). Specifically, a line/rake is created at the nozzle outlet, which is to obtain the concentration information near the nozzle. As the proportion of the discrete phase in the continuous phase is very low and the inter-particle interaction can be ignored, when injecting the particles, the discrete phase is injected in one of the carrier gas flow inlets (*e.g.*, left CGF inlet) to get the concentration profile on the line/rake. The concentration profile from the other inlet can be obtained accordingly by taking symmetry along the centerline. The overlapping area of the original profile and symmetrically operated profile can be used to evaluate the degree of mixing. A mixing index was defined below:

Mixing index = Area of overlap area / Area of one of the concentration profile

$$\textbf{\textit{Mixing index}} = \frac{\int C_m(x)dx}{\int C_1(x)dx} = \frac{\int C_m(x)dx}{\int C_2(x)dx} \quad (7)$$

Where the $C_m(x)$ is the local concentration of the mixed portion (the region where there is a mixing of two aerosols) at a specific location x, and the $C_1(x)$ and $C_2(x)$ are the local concentration of aerosol 1 and aerosol 2 at a specific location x, respectively. Under a completely unmixed case, the mixing index is 0, while it becomes 1 for a completely mixed scenario. It is noted that this mixing index describes the degree of mixing near the nozzle, while further mixing may also occur when aerosols leave the nozzle in the air or on a substrate during the ink phase.

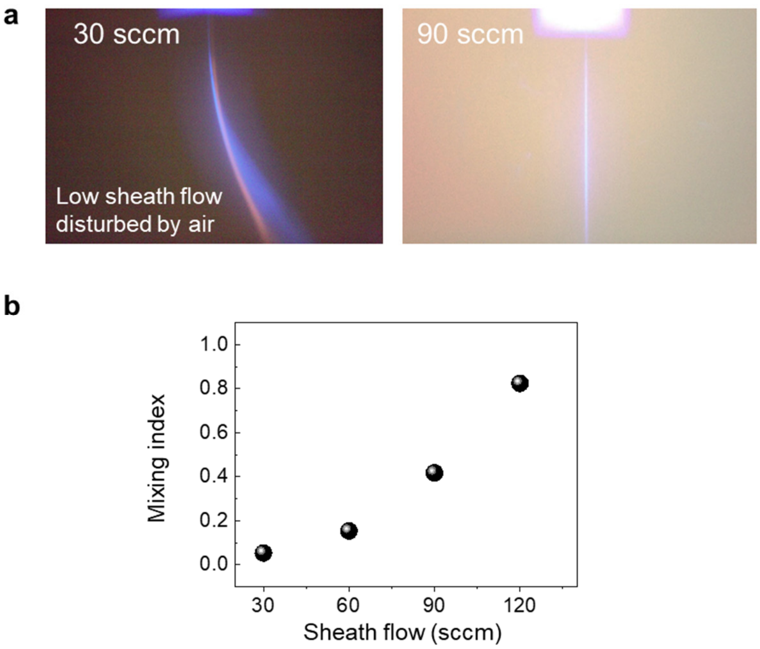

Fig. S22. Comparison of aerosol mixing with CFD simulation. **a**, Fast camera image of blue and red ink aerosols under the sheath flow of 30 sccm (left) and 90 sccm (right). **b**, CFD simulated the mixing index of HTCP mixed aerosols of blue and red inks near the jetting nozzle. The mixing index was obtained by calculating the mixed portion of ink aerosols (*e.g.*, ink 1) divided by the entire aerosol concentration (under a completely unmixed case, the mixing index is zero while it becomes 1 for a completely mixed scenario).

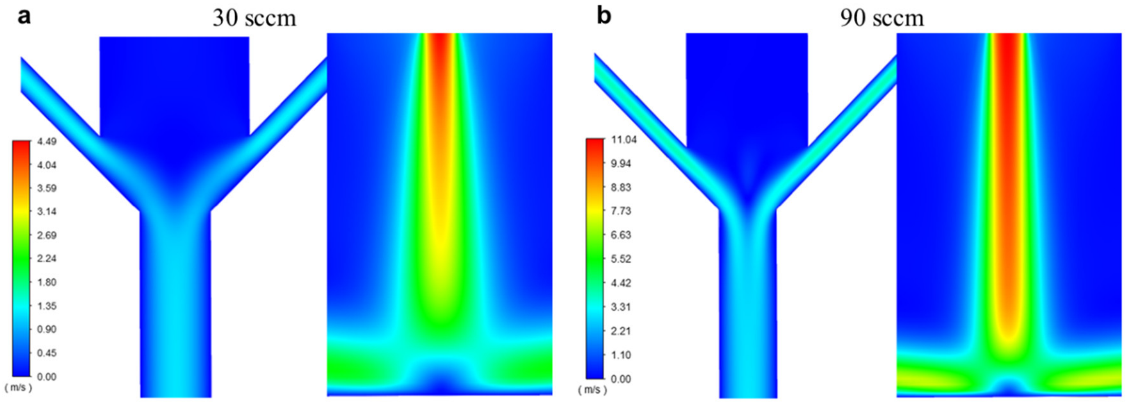

Fig. S23. CFD simulation on the velocity profile of low sheath flow condition (**a**) and high sheath flow condition (**b**).

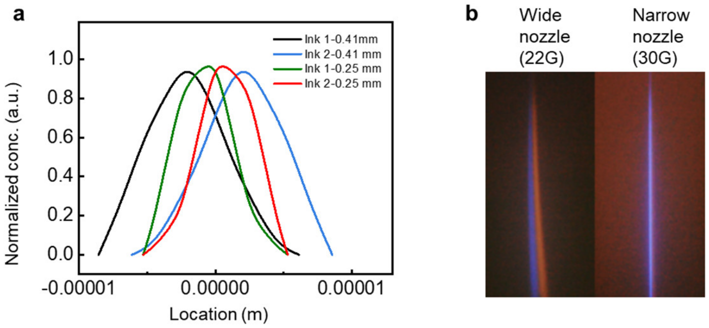

Fig. S24. Effect of aerosol mixing and printing. **a**, CFD simulation on aerosol mixing with different nozzle dimensions (diameters). **b**, Fast camera image of aerosols with different nozzle sizes (22G and 30G).

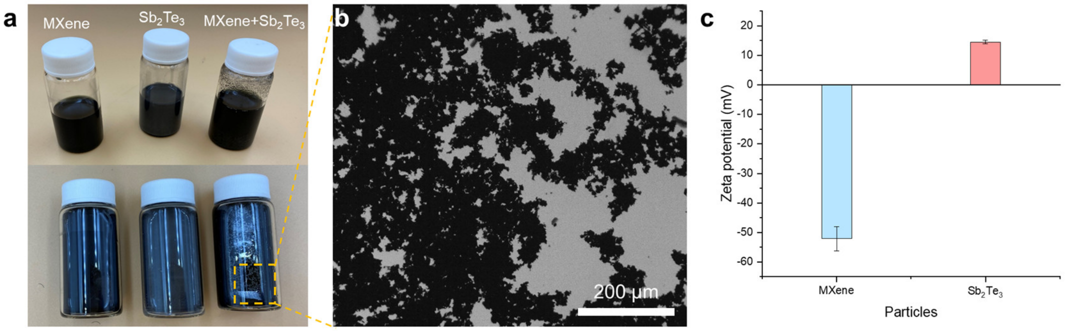

Fig. S25. Ink incompatibility by conventional mixing due to charge mismatch. **a**, Photographic images of MXene ink, Sb_2Te_3 ink, and the mixed ink of MXene and Sb_2Te_3. **b**, Optical microscopy image showing large aggregates caused by charge mismatched MXene and Sb_2Te_3. **c**, Zeta potential of colloidal MXene and Sb_2Te_3, showing negative and positive surface potential values, respectively. Error bars represent standard deviations from 3 experimental replicates.

As shown in photographic images, large aggregates started to form (15 s ~ 1 min) once Sb_2Te_3 and MXene were mixed together, where these particle aggregates can be seen at the wall of glass vials as well as the solution/air interface. The optical microscopic image indicated the size of these aggregates in the range of 30 to 1000 μm, which inevitably caused undesired effects for the printing process. As evidenced by zeta potential analyses (at a neutral pH environment), the MXene shows a strongly negative surface charge while Sb_2Te_3 shows a moderately positive charge.

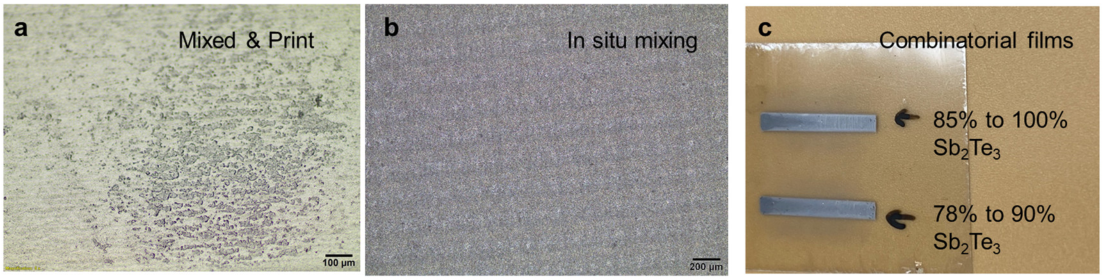

Fig. S26. Comparison of HTCP with conventional ink mixing. **a,b**, Optical microscopy images of printed films made from conventional mixing of MXene and Sb_2Te_3 (**a**) and HTCP mixing of MXene and Sb_2Te_3 (**b**). **c**, Photographic images of two combinatorial films of MXene and Sb_2Te_3.

Optical microscopy was used to further evaluate the morphology of printed combinatorial materials from different mixing mechanisms. The optical microscopy image of MXene and Sb_2Te_3 via conventional mixing reveals some materials loss after thermal annealing which is likely due to the inherently structural inhomogeneity that induces unevenly distributed local strain/stress [13-15]. By contrast, the well-mixed nanocomposite films of MXene and Sb_2Te_3 via HTCP demonstrate a much more uniform morphology thanks to the *in situ* mixing of MXene aerosols and Sb_2Te_3 aerosols on the fly. Therefore, combinatorial films of MXene and Sb_2Te_3 with various mixing ratios were successfully fabricated, where the ink flow rate percentages (*i.e.*, aerosol percentages) of Sb_2Te_3 were noted.

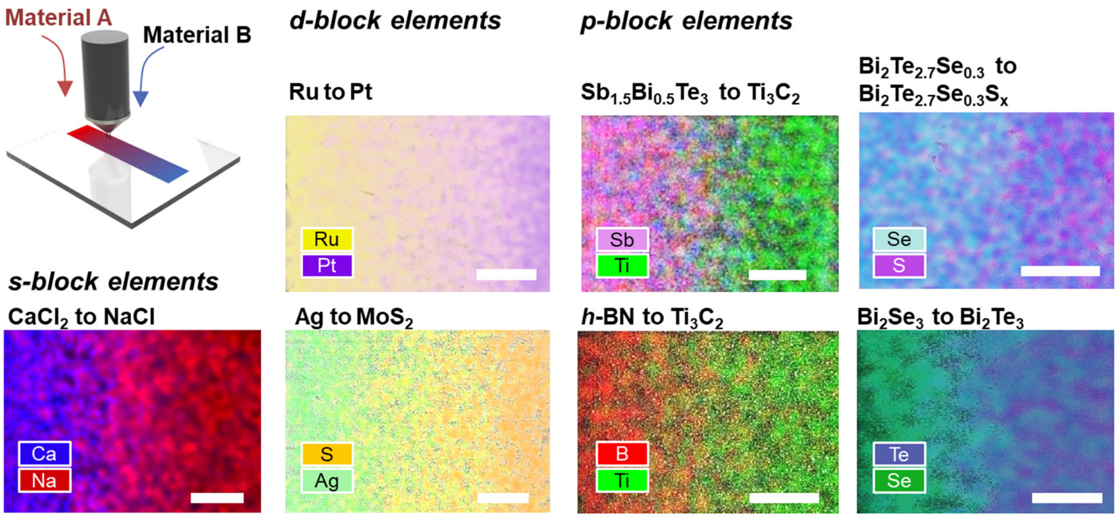

Fig. S27. Elemental analyses of various type of combinatorial material libraries that contains elements from s-block, d-block, and p-block groups of the periodic table. Scale bar: 300 µm.

To analyze the elemental distribution of various combinatorial films, energy-dispersive X-Ray spectroscopy (EDS) and X-ray fluorescence imaging (XRF) techniques are used to map the containing elements with spatial resolution. These combinatorial materials contain elements from the s-block, d-block, and p-block groups of the periodic table.

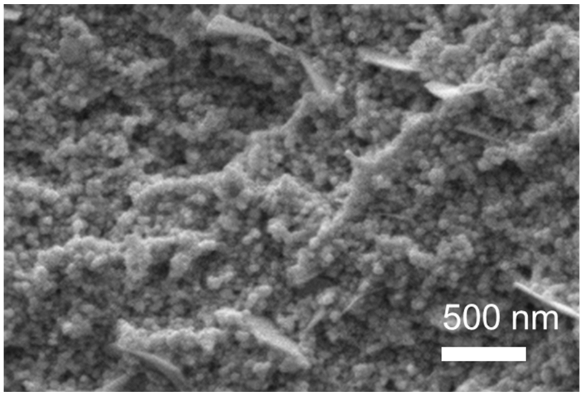

Fig. S28. SEM image of a cross-sectional view of nanocomposite of Bi_2Te_3 and Ag particles.

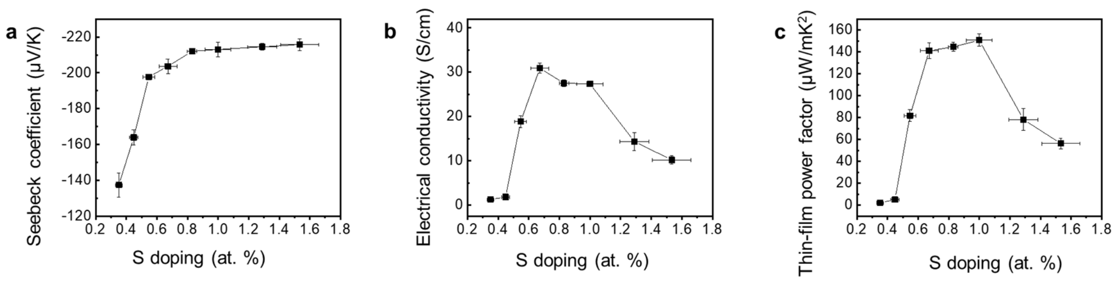

Fig. S29. Combinatorial sulfur doping of n-type $Bi_2Te_{2.7}Se_{0.3}$. **a**, Seebeck coefficient vs. sulfur doping concentrations. **b**, Electrical conductivity vs. sulfur doping concentrations. **c**, Thin-film power factor (PF) vs. sulfur doping concentrations. Error bars represent standard deviations from 2 experimental replicates (Y error bars) and 6 experimental replicates (X error bars).

As shown in the above figure, wavelength-dispersive spectroscopy (WDS) was used to map the sulfur doping concentration (at. %) on the combinatorial doped $Bi_2Te_{2.7}Se_{0.3}$ film. The Seebeck coefficient and electrical conductivity were measured across the entire sample locations using a custom-built high-throughput probing system at the University of Notre Dame.

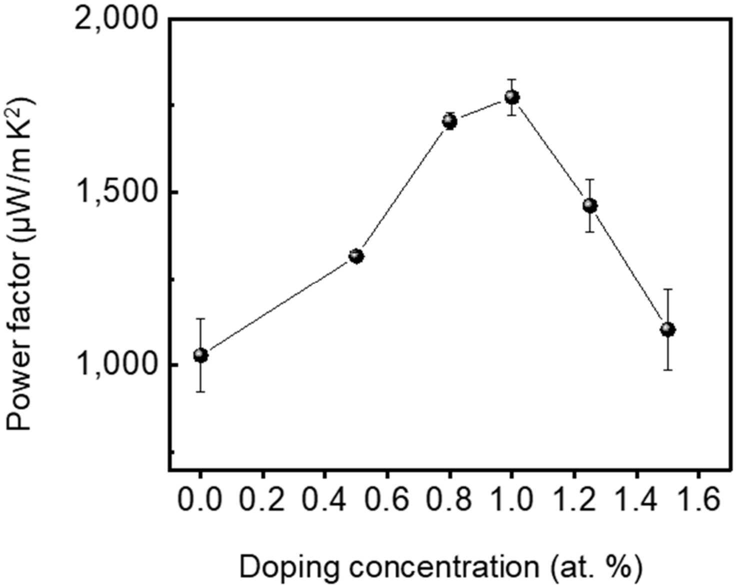

Fig. S30. The thermoelectric power factor of extrusion printed $Bi_2Te_{2.7}Se_{0.3}$ samples with various sulfur doping concentrations. The error bar represents the standard deviation of the three samples made under each doping concentration.

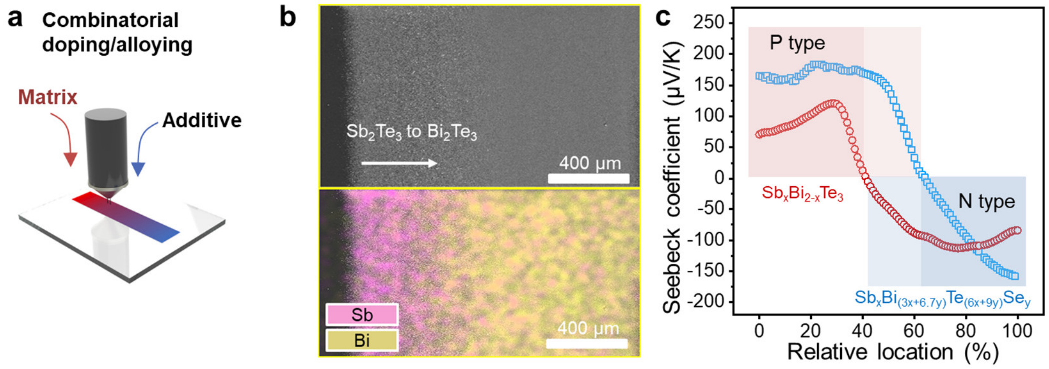

Fig. S31. Combinatorial doping/alloying for active tuning of the transport behaviors of charge carriers. **a**, Schematics of combinatorial doping/alloying. **b**, Combinatorial $Sb_xBi_{2-x}Te_3$ was used as an example by changing the Sb/Bi ratio with SEM/EDS images. **c**, Seebeck coefficient measurements indicate the active tuning of carrier concentration as well as the carrier type.

From a fundamental perspective, we hypothesize the combinatorial feature of HTCP may provide a valuable tool for the mechanistic study of the compositional effect on charge carrier transport behaviors. Here, the Seebeck coefficient (referring to the process of the thermodynamic chemical potential of charge carriers[16]) is selected as one metric for assessing the transport property of charge carriers in combinatorial alloys. Control of the major charge carriers from holes to electrons was demonstrated by printing an alloy film consisting of semiconducting p- and n-type nanoplates. During the HTCP process, the antimony content of ternary $Sb_xBi_{2-x}Te_3$ and quaternary $Sb_xBi_{(0.3x+6.7y)}Te_{(2x+9y)}Se_y$ alloys decreased along the gradient film, leading to a significant change in the Seebeck coefficient from positive to negative region. The Sb/Bi ratio tuned by HTCP can significantly change the Seebeck coefficient due to the controllable modulation of defect chemistry[17], revealing effective control of the major charge carrier concentration as well as carrier type from holes to electrons. For example, the Seebeck coefficient of ternary $Sb_xBi_{2-x}Te_3$ first increases and then decreases to the negative region, and this is an indication of decreasing hole concentration of the alloy[17].

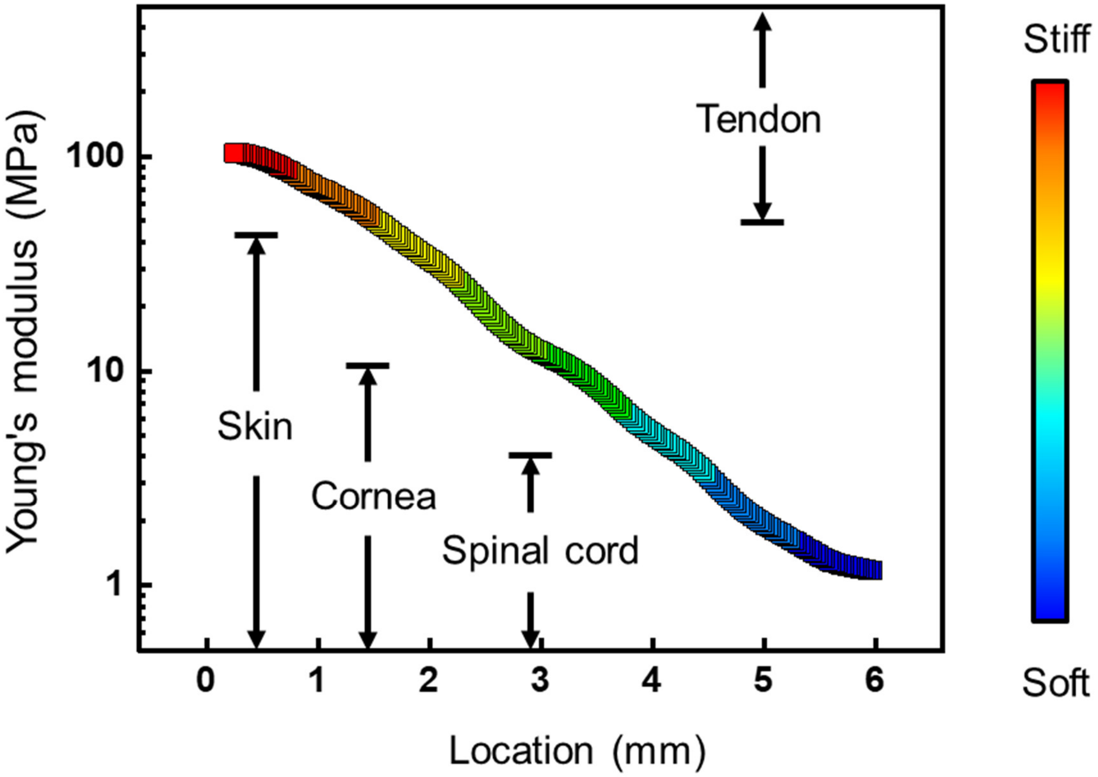

Fig. S32. Young's modulus of functionally graded polyurethane films in comparison with that of biomaterials[18].

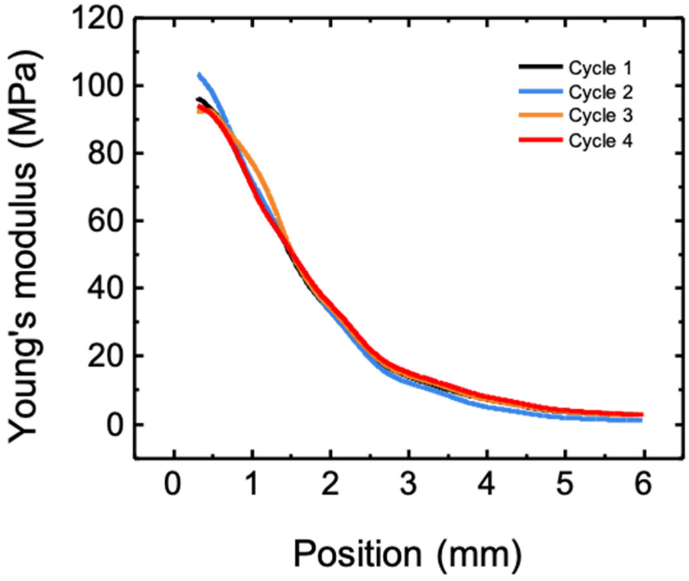

Fig. S33. Young's modulus of functionally graded polyurethane films vs sample locations measured during 4 repeated stretching-releasing cycles with up to 50% strain (stretching ratio).

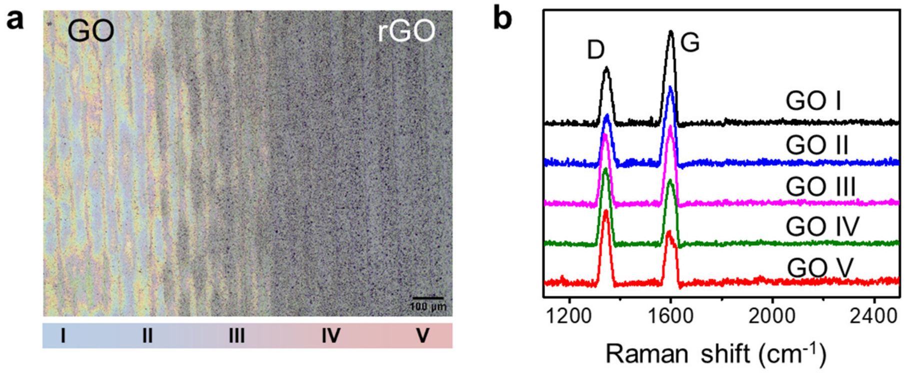

Fig. S34. Combinatorial reaction of GO with reducing agent. **a**, Optical microscopic image of GO/rGO gradient film during a combinatorial reaction, showing dark spots that emerge upon introducing an increasing amount of reducing agent. **b**, Raman spectra demonstrate the change of the D band and G band of GO at different locations.

Supplemental Tables

Table S1: Comparison of typical processing parameters of different combinatorial fabrication methods.

	Sputtering	Inkjet	Extrusion	HTCP (this work)
**Sample library**	Dense	Dense	Dense	Dense
**In-situ ink mixing and fast modulation**	-	Maybe	No	Yes
**External mixer**	Not needed	Maybe	Needed	Not needed
**Shape control**	No	Yes	Yes	Yes
**Fabrication in 3D**	No	No	Yes	Yes
**Minimal resolution**	Material- and processing-dependent	10 µm	100 µm	~20 µm (xy-axis), 100 nm (z-axis)

Table S2. Typical ink formulation of nanoparticle inks. PVP: polyvinylpyrrolidone; SDBS: sodium dodecylbenzenesulfonate; DMF: dimethylformamide.

**Materials**	**Solvent**	**Co-solvent**	**Mass percentage**	**Surfactants/Additives**
Graphene	Water	Ethylene glycol	0.2 to 1.0 wt. %	Graphene quantum dots
Bi_2Te_3	Water	Ethylene glycol	3.0 to 10.0 wt. %	PVP
$Sb_{1.5}Bi_{0.5}Te_3$	Water	Ethylene glycol	6.0 to 10.0 wt. %	PVP
$Bi_2Te_3Se_{0.3}$	Water	Ethylene glycol	6.0 to 10.0 wt. %	PVP
Doped $Bi_2Te_3Se_{0.3}$	Water	Ethylene glycol	4 wt. %	PVP and 1-thioglycerol
Ag nanoparticles	Water	Ethylene glycol	2 wt. %	PVP
Ag nanowires	DMF	-	1 wt. %	PVP
Molybdenum disulfide (MoS_2)	Terpineol	Cyclohexanone	3.0 to 3.85 wt. %	Ethyl cellulose
MXene (Ti_3C_2)	Water	Ethylene glycol	1 wt. %	-
Polystyrene	Water	Ethylene glycol	1 wt. %	SDBS
Te nanowire	Water	Ethylene glycol	1 wt. %	PVP

Table S3. Aerosol jet printing parameters of combinatorial thermoelectric films.

**Parameters**	**Values**
Nozzle nominal I.D. (μm)	159 (30G)-413 (22G), ~1700 (glass nozzle)
Ink flow rate (sccm)	0-30
Sheath gas flow rate (sccm)	20-120
Platen temperature (°C)	85
Print speed (mm/s)	1-5

Table S4. Aerosol jet printing parameters of graphene films showing consistent deposition thickness.

Parameters	Sample 1	Sample 2	Sample 3	Sample 4
Nozzle size	22G	22G	22G	22G
Ink flow rate (sccm)	14	14	14	14
Sheath gas flow rate (sccm)	50	50	50	50
Ultrasonic atomizing voltage (V)	30	30	30	30
Platen temperature (°C)	85	85	85	85
Print speed (mm/s)	1.0	1.0	1.0	1.0
Deposition thickness (μm)	1.27	1.21	1.27	1.17

Table S5. Aerosol jet printing of graphene films with different printing speeds, exclusively showing uncertainty less than 10.0 %. Average deposition thickness, population/sample standard deviation, and uncertainty are calculated from 4 experimental replicates.

Parameters	Printing speed #1	Printing speed #2	Printing speed #3	Printing speed #4
Batch 1 (μm)	1.27	0.65	0.29	0.29
Batch 2 (μm)	1.21	0.7	0.32	0.25
Batch 3 (μm)	1.27	0.57	0.33	0.28
Batch 4 (μm)	1.17	0.59	0.31	0.26
Print speed (mm/s)	1.0	2.0	3.0	4.0
Sheath gas flow rate (sccm)	50	50	50	50
Ultrasonic atomizing voltage (V)	30	30	30	30
Platen temperature (°C)	85	85	85	85
Ink flow rate (sccm)	14	14	14	14
Average thickness (μm)	1.230	0.6275	0.3125	0.2700
Sample standard deviation (μm)	0.04899	0.05909	0.01708	0.01826
Population standard deviation (μm)	0.04243	0.05117	0.01479	0.01581
Relative uncertainty	3.98%	9.42%	5.47%	6.76%

Table S6. Parameter table of CFD simulation.

Characteristics	Value
Operating pressure	101,325 Pa
Operating temperature	288.16 K
Material of continuous phase	Nitrogen
Material type of discrete phase	Inert
Density of discrete phase	1000 kg/m^3
Diameter of discrete phase	3×10^{-6} m
Total flow rate of discrete phase	1×10^{-8} kg/s
Velocity of CGF inlet	0.0184 m/s
Velocity of ShGF inlet	0.6366, 1.2732, 1.9099, 2.5465 m/s
Size of nozzle outlet	0.25/0.41 mm

Table S7. Comparison of printed thermoelectric materials on their thermoelectric power factors. *The thermal conductivity data are not always readily available for printed TE materials, and thus we here focus on Seebeck coefficient (S), electrical conductivity (σ), and power factor (PF=$S^2\sigma$).

Ref.	Materials	Fabrication	S (μV/K)	σ (S/cm)	PF* μW/(m·K^2)
This work	$Bi_2Te_{2.7}Se_{0.3}$	Extrusion printing	-186	513	1,774
[19]	$Bi_2Te_{2.8}Se_{0.2}$	Screen printing	-126	310	490
[20]	Bi_2Te_3/PEDOT	Screen printing	-138	73	138.6
[21]	Bi_2Te_3/epoxy	Extrusion printing	-157	61	150
[22]	Bi_2S_3/PANI	Drop casting	-42.8	0.4	0.07
[23]	Poly[Kx(Ni-ett)] /PVDF	Inkjet printing	-44.9	2.1	0.4
[24]	V_2O_5/PEDOT	Inkjet printing	-350	0.16	2
[25]	Bi_2Te_3/Se/epoxy	Extrusion printing	-170	96	277
[26]	$Bi_2Te_{2.7}Se_{0.3}$	Extrusion printing	-120	500	720
[27]	$Bi_2Te_{2.7}Se_{0.3}$	Aerosol jet printing	-163	270	730
[28]	$TiS_2(HA)_x$	Inkjet printing	-70	430	211

Supplementary References

1 Zeng, M. *et al.* Aqueous exfoliation of graphite into graphene assisted by sulfonyl graphene quantum dots for photonic crystal applications. *ACS Appl. Mater. Interfaces* **9**, 30797-30804 (2017).

2 Zeng, M. *et al.* Colloidal Nanosurfactants for 3D Conformal Printing of 2D van der Waals Materials. *Adv. Mater.* **32**, e2003081, doi:10.1002/adma.202003081 (2020).

3 Saeidi-Javash, M. *et al.* All-Printed MXene–Graphene Nanosheet-Based Bimodal Sensors for Simultaneous Strain and Temperature Sensing. *ACS Appl. Electron. Mater.* **3**, 2341-2348, doi:10.1021/acsaelm.1c00218 (2021).

4 Wyatt, B. C. *et al.* High-temperature stability and phase transformations of titanium carbide (Ti3C2Tx) MXene. *J. Phys.: Condens. Matter* **33**, 224002, doi:10.1088/1361-648X/abe793 (2021).

5 Yan, Y. G., Martin, J., Wong-Ng, W., Green, M. & Tang, X. F. A temperature dependent screening tool for high throughput thermoelectric characterization of combinatorial films. *Rev. Sci. Instrum.* **84**, 115110, doi:10.1063/1.4830295 (2013).

6 Martin, J. Protocols for the high temperature measurement of the Seebeck coefficient in thermoelectric materials. *Meas. Sci. Technol.* **24**, 085601, doi:10.1088/0957-0233/24/8/085601 (2013).

7 Blaber, J., Adair, B. & Antoniou, A. Ncorr: Open-Source 2D Digital Image Correlation Matlab Software. *Experimental Mechanics* **55**, 1105-1122, doi:10.1007/s11340-015-0009-1 (2015).

8 Chen, G., Gu, Y., Tsang, H., Hines, D. R. & Das, S. The Effect of Droplet Sizes on Overspray in Aerosol-Jet Printing. *Adv. Eng. Mater.* **20**, 1701084, doi:https://doi.org/10.1002/adem.201701084 (2018).

9 Poiseuille, J. L. M. *Recherches sur les causes du mouvement du sang dans les vaisseaux capillaires*. Vol. 7 (Impr. royale, 1839).

10 Akhatov, I. S., Hoey, J. M., Swenson, O. F. & Schulz, D. L. Aerosol focusing in micro-capillaries: Theory and experiment. *J. Aerosol Sci* **39**, 691-709, doi:https://doi.org/10.1016/j.jaerosci.2008.04.004 (2008).

11 Wang, X., Gidwani, A., Girshick, S. L. & McMurry, P. H. Aerodynamic Focusing of Nanoparticles: II. Numerical Simulation of Particle Motion Through Aerodynamic Lenses. *Aerosol Sci. Technol.* **39**, 624-636, doi:10.1080/02786820500181950 (2005).

12 Wang, X., Kruis, F. E. & McMurry, P. H. Aerodynamic Focusing of Nanoparticles: I. Guidelines for Designing Aerodynamic Lenses for Nanoparticles. *Aerosol Sci. Technol.* **39**, 611-623, doi:10.1080/02786820500181901 (2005).

13 Guo, J., Sun, A., Chen, X., Wang, C. & Manivannan, A. Cyclability study of silicon–carbon composite anodes for lithium-ion batteries using electrochemical impedance spectroscopy. *Electrochim. Acta* **56**, 3981-3987, doi:https://doi.org/10.1016/j.electacta.2011.02.014 (2011).

14 Scherer, G. W. Sintering inhomogeneous glasses: Application to optical waveguides. *J. Non-Cryst. Solids* **34**, 239-256, doi:https://doi.org/10.1016/0022-3093(79)90039-5 (1979).

15 Wang, J. *et al.* Surface stress effect in mechanics of nanostructured materials. *Acta Mechanica Solida Sinica* **24**, 52-82, doi:10.1016/S0894-9166(11)60009-8 (2011).

16 Wang, Y. *et al.* First-principles thermodynamic theory of Seebeck coefficients. *Physical Review B* **98**, 224101, doi:10.1103/PhysRevB.98.224101 (2018).

17 Witting, I. T. *et al.* The Thermoelectric Properties of Bismuth Telluride. *Advanced Electronic Materials* **5**, 1800904, doi:https://doi.org/10.1002/aelm.201800904 (2019).

18 McKee, C. T., Last, J. A., Russell, P. & Murphy, C. J. Indentation versus tensile measurements of Young's modulus for soft biological tissues. *Tissue Engineering Part B: Reviews* **17**, 155-164 (2011).

19 Varghese, T. *et al.* High-performance and flexible thermoelectric films by screen printing solution-processed nanoplate crystals. *Scientific Reports* **6**, 33135, doi:10.1038/srep33135

https://www.nature.com/articles/srep33135#supplementary-information (2016).

20 We, J. H., Kim, S. J. & Cho, B. J. Hybrid composite of screen-printed inorganic thermoelectric film and organic conducting polymer for flexible thermoelectric power generator. *Energy* **73**, 506-512, doi:https://doi.org/10.1016/j.energy.2014.06.047 (2014).

21 Chen, A., Madan, D., Wright, P. K. & Evans, J. W. Dispenser-printed planar thick-film thermoelectric energy generators. *Journal of Micromechanics and Microengineering* **21**, 104006, doi:10.1088/0960-1317/21/10/104006 (2011).

22 Wang, Y., Liu, G., Sheng, M., Yu, C. & Deng, Y. Flexible thermopower generation over broad temperature range by PANI/nanorod hybrid-based p–n couples. *Journal of Materials Chemistry A* **7**, 1718-1724, doi:10.1039/C8TA11008E (2019).

23 Jiao, F. *et al.* Inkjet-printed flexible organic thin-film thermoelectric devices based on p- and n-type poly(metal 1,1,2,2-ethenetetrathiolate)s/polymer composites through ball-milling. *Philosophical Transactions of the Royal Society A: Mathematical, Physical and Engineering Sciences* **372**, 20130008, doi:doi:10.1098/rsta.2013.0008 (2014).

24 Ferhat, S. *et al.* Organic thermoelectric devices based on a stable n-type nanocomposite printed on paper. *Sustainable Energy & Fuels* **2**, 199-208, doi:10.1039/C7SE00313G (2018).

25 Madan, D. *et al.* Enhanced Performance of Dispenser Printed MA n-type Bi2Te3 Composite Thermoelectric Generators. *ACS Appl. Mater. Interfaces* **4**, 6117-6124, doi:10.1021/am301759a (2012).

26 Kim, F. *et al.* 3D printing of shape-conformable thermoelectric materials using all-inorganic Bi2Te3-based inks. *Nature Energy* **3**, 301-309, doi:10.1038/s41560-017-0071-2 (2018).

27 Saeidi-Javash, M., Kuang, W., Dun, C. & Zhang, Y. 3D Conformal Printing and Photonic Sintering of High-Performance Flexible Thermoelectric Films Using 2D Nanoplates. *Adv. Funct. Mater.* **29**, 1901930, doi:10.1002/adfm.201901930 (2019).

28 Ferhat, S. *et al.* Flexible thermoelectric device based on TiS2(HA)x n-type nanocomposite printed on paper. *Org. Electron.* **68**, 256-263, doi:https://doi.org/10.1016/j.orgel.2019.02.031 (2019).
